# Impact of elexacaftor/tezacaftor/ivacaftor on lung function, nutritional status, pulmonary exacerbation frequency and sweat chloride in people with cystic fibrosis: real-world evidence from the German CF Registry

**DOI:** 10.1016/j.lanepe.2023.100690

**Published:** 2023-07-28

**Authors:** Sivagurunathan Sutharsan, Stefanie Dillenhoefer, Matthias Welsner, Florian Stehling, Folke Brinkmann, Manuel Burkhart, Helmut Ellemunter, Anna-Maria Dittrich, Christina Smaczny, Olaf Eickmeier, Matthias Kappler, Carsten Schwarz, Sarah Sieber, Susanne Naehrig, Lutz Naehrlich, German CF Registry of the Mukoviszidose e.V. and participating CF sites, Klaus Tenbrock, Claus Pfannenstiel, Dirk Steffen, Jochen Meister, Britta Welzenbach, Anette Scharschinger, Markus Kratz, Maike Pincus, Tobias Tenenbaum, Mirjam Stahl, Kerstin Landwehr, Stefanie Dillenhöfer, Hans Kössel, Petra Kaiser, Manfred Käding, Simone Stolz, Stefan Blaas, Jutta Hammermann, Monika Gappa, Antje Schuster, Dana Spittel, Sabine Zirlik, Sabina Schmitt, Florian Stehling, Sivagurunathan Sutharsan, Joachim Bargon, Malte Cremer, Christina Smaczny, Sebastian Fähndrich, Andrea Heinzmann, Lutz Nährlich, Stefan Kuhnert, Sebastian Schmidt, Bettina Wollschläger, Anna Nolde, Inka Held, Wolfgang Kamin, Felix C. Ringshausen, Anna-Maria Dittrich, Sabine Wege, Olaf Sommerburg, Norbert Geier, Sara Lisa Fleser, Heinrike Wilkens, Helmut Ellemunter, Michael Lorenz, Paul Vöhringer, Martin Schebek, Christian Timke, Ingrid Bobis, Thomas Nüßlein, Doris Dieninghoff, Ernst Rietschel, Bastian Klinkhammer, Freerk Prenzel, Alexandra Wald, Axel Kempa, Folke Brinkmann, Eva Lücke, Ines Adams, Krystyna Poplawska, Simone Lehmkühler, Monika Bauck, Anne Pfülb, Rainald Fischer, Gudrun Schopper, Susanne Nährig, Matthias Griese, Jörg Grosse, Peter Küster, Birte KinderHolger Köster, Susanne Büsing, Margarethe Pohl, Carsten Schwarz, Andreas Artlich, Alexander Kiefer, Manfred Ballmann, Nikola Gjorgjevski, Markus A. Rose, Friederike Ruf, Rolf Mahlberg, Wolfgang Thomas, Ute Graepler, Sebastian Bode, hilipp Meyn, Josef Rosenecker, Cordula Koerner, Klaus-Michael Keller, Tina Teßmer, Helge Hebestreit, Gerhild Lohse

**Affiliations:** a Department of Pulmonary Medicine, University Hospital Essen-Ruhrlandklinik, Adult Cystic Fibrosis Center, University of Duisburg-Essen, Essen, Germany; b Department of Pediatric Pulmonology, Cystic Fibrosis Center, University Children's Hospital of Ruhr University Bochum at St. Josef-Hospital, Bochum, Germany; c Pediatric Pulmonology and Sleep Medicine, Children's University Hospital Essen, University of Duisburg-Essen, Essen, Germany; d Department of Pediatric Pneumology & Allergology, The University of Lübeck, University Medical Center Schleswig-Holstein, Campus Centrum Lübeck, Member of Airway Research Center North (ARCN) of the German Center of Lung Research (DZL), Lübeck, Germany; e Mukoviszidose Institut gGmbH (MI), Bonn, Germany; f Medical University of Innsbruck, Cystic Fibrosis Centre Innsbruck, Innsbruck, Austria; g Department of Paediatric Pneumology, Allergology and Neonatology, Hannover Medical School, Hannover, Germany; h University Hospital Frankfurt/Main, Goethe University, Pneumology and Allergology, Christiane Herzog CF Center Frankfurt/Main, Frankfurt/Main, Germany; i Pediatric Allergology, Pulmonology & Cystic Fibrosis, Christiane Herzog CF Center- Frankfurt a.M., University Hospital Frankfurt a.M., Germany; j Department of Pediatrics, Dr. von Hauner Children's Hospital, University Hospital, LMU Munich, Germany; k Division Cystic Fibrosis, HMU-Health and Medical University Potsdam, Clinic Westbrandenburg, Potsdam, Germany; l STAT-UP Statistical Consulting & Data Science GmbH, Munich, Germany; m Department of Internal Medicine V, Cystic Fibrosis Center for Adults, University Hospital, Ludwig Maximilian University (LMU) Munich, Germany; n Department of Pediatrics, Justus-Liebig-University Giessen, Giessen, Germany; o Universities of Giessen and Marburg Lung Center (UGMLC), German Center for Lung Research (DZL), Giessen, Germany; p Biomedical Research in Endstage and Obstructive Lung Disease Hannover (BREATH), Member of the German Center for Lung Research (DZL), Germany

**Keywords:** Cystic fibrosis, Elexacaftor/tezacaftor/ivacaftor, Lung function, Body mass index, Pulmonary exacerbation, Sweat chloride, Real-world evidence

## Abstract

**Background:**

Treatment with elexacaftor/tezacaftor/ivacaftor (ETI) improves multiple clinical outcomes in people with cystic fibrosis (pwCF) with at least one F508del allele. This study evaluated the real-world impact of ETI on lung function, nutritional status, pulmonary exacerbation frequency, and sweat chloride concentrations in a large group of pwCF.

**Methods:**

This observational cohort study used data from the German CF Registry for pwCF who received ETI therapy and were followed up for a period of 12 months.

**Findings:**

The study included 2645 pwCF from 67 centres in Germany (mean age 28.0 ± 11.5 years). Over the first year after ETI was initiated, percent predicted forced expiratory volume in 1 s (ppFEV_1_) increased by 11.3% (95% confidence interval [CI] 10.8–11.8, p < 0.0001), body mass index (BMI) *z*-score increased by 0.3 (95% CI 0.3–0.4, p < 0.0001) in individuals aged 12 to <18 years and BMI in adults increased by 1.4 kg/m^2^ (95% CI 1.3–1.4, p < 0.0001), pulmonary exacerbations decreased by 75.9% (p < 0.0001) and mean sweat chloride concentration decreased by 50.9 mmol/L (95% CI –52.6, −49.3, p < 0.0001). Improvements in ppFEV_1_ over the first year of therapy were greater in pwCF who had not previously received cystic fibrosis transmembrane conductance regulator (CFTR) modulator therapy (12.6% [95% CI 11.9–13.4] vs. 9.7% [95% CI 9.0–10.5] in those with prior CFTR modulator treatment.

**Interpretation:**

These real-world data are consistent with the findings of randomised clinical trials, and support the use of ETI as a highly effective treatment option for pwCF who have at least one F508del allele.

**Funding:**

None.

**Research in context**

**Evidence before this study**

We searched PubMed for articles using the terms “elexacaftor/tezecaftor/ivacaftor” or “clinical trial” or CFTR modulator” or “real world evidence” and “cystic fibrosis” from database inception to April 17, 2023, with no language restrictions. A number of randomised clinical trials have documented rapid and sustained improvements in lung function and nutritional status, and a reduction in pulmonary exacerbations, during triple combination cystic fibrosis transmembrane conductance regulator (CFTR) modulator therapy with elexacaftor/tezacaftor/ivacaftor (ETI) in people with cystic fibrosis (pwCF) carrying at least one F508del mutation. Current CFTR modulator therapies, such as elexacaftor–tezacaftor–ivacaftor, have transformed cystic fibrosis care.

**Added value of this study**

This real-world evidence from the German CF Registry provides important insights into the impact of ETI therapy in a large heterogeneous cohort of adolescent and adult pwCF treated for at least one year in clinical practice.

**Implications of all the available evidence**

The consistency of real-world evidence with clinical trial data supports the use and effectiveness of ETI in pwCF. The observed increases in body mass index during ETI therapy highlight the need for careful monitoring of body weight and nutritional status, with associated modifications to nutritional management and regular monitoring of cardiovascular risk, during the long-term use of this treatment in clinical practice.

## Introduction

Cystic fibrosis (CF) is a multisystem, life-limiting, autosomal recessive genetic disease that affects more than 7000 children and adults in Germany, and approximately 100,000 people worldwide.^1^, ^2^, ^3^, ^4^ Clinical manifestations are due to mutations in the cystic fibrosis transmembrane conductance regulator (CFTR) gene, and include liver, pancreatic, and gastrointestinal symptoms, but pulmonary disease is the major cause of morbidity and mortality.^5^^,^^6^ In Germany, the median survival age for people with CF (pwCF) has increased to 54 years due to progress in treatment so that the number of adult pwCF has exceeded the paediatric cohort since 2008.^5^^,^^6^

The new standard of treatment for CF is CFTR modulator therapy, including CFTR potentiators (e.g., ivacaftor) and CFTR correctors (e.g., elexacaftor, tezacaftor, lumacaftor). These agents (mostly used in combination) effectively improve impaired CFTR function to improve lung function, nutritional status and quality of life, and decrease sweat chloride concentrations and exacerbation frequency in pwCF carrying at least one F508del mutation.^7^, ^8^, ^9^, ^10^, ^11^

In August 2020, the triple combination of elexacaftor/tezacaftor/ivacaftor (ETI) was approved in Europe for the treatment of pwCF aged ≥12 years and F508del homozygous or heterozygous in combination with a minimal function mutation. Approval has since been extended to all heterozygous pwCF aged ≥12 years, and then to all pwCF aged ≥6 years with at least one F508del mutation.

Since its approval in Germany in August 2020, the use of ETI has become widespread, but there are limited data on the use of ETI in the real-world setting. This observational cohort study utilised data from the German CF Registry of the Mukoviszidose e.V.^3^ to evaluate the magnitude and durability of the clinical impact of triple combination therapy with ETI in a large cohort in pwCF who have at least one F508del CFTR mutation.

## Methods

### Study design, population and treatment

This study evaluated encounter-based documentation from the German CF registry^3^ from 1 January 2019 to 7 February 2023. Approximately 80% of all pwCF in Germany provide annual data for the German CF registry (2021: 6776 pwCF; 87 centres), of which encounter-based documentation is established for 90% of all pwCF from 67 centers.^3^ The study was approved by the Ethics Committee of the Justus-Liebig-University, Giessen, Germany (AZ24/19). Written informed consent was obtained from all participants and minor(s)' legal guardian/next of kin.

From the 67 centres with encounter-based documentation, all pwCF eligible for ETI (F508del/any other mutation) who were aged ≥12 years at the start of ETI, had an ETI therapy duration of ≥12 months, and no history of transplantation (any organ) were included. Those receiving CFTR modulators in the context of clinical trials were excluded.

ETI therapy with interruptions of up to 90 days was considered to be continuous therapy. If ETI therapy was restarted after a break of >90 days, only the first period of therapy was included in the analysis. If the end date of ETI therapy was missing, it was assumed that therapy has not yet ended. Examinations within the first 4 weeks after initiation of ETI were not considered to allow development of the full effect of ETI therapy, as described previously.^8^ We identified a consistent subgroup pwCF who had complete data for ppFEV1 and BMI (divided in BMI over 18 years and BMI z-score for <18 years) across all four quarters before and after initiating ETI therapy. This subgroup can serve as a validation cohort for the larger group of pwCF with incomplete data.

### Study parameters

Data were obtained for lung function (measured according to American Thoracic Society/European Respiratory Society criteria and analysed using Global Lung Function Initiative equations for Caucasians) and body mass index (BMI; reported as *z*-scores for adolescents^12^ and absolute values for adults). Sweat chloride levels were measured at least once in the last year before starting ETI and during the first year of ETI treatment. An exacerbation was defined as the need for additional oral or intravenous antibiotic treatment as indicated by at least two of the following: change in sputum volume or colour; increased cough; increased malaise, fatigue or lethargy; anorexia or weight loss; ≥10% decrease in pulmonary function; radiographic changes; and/or increased dyspnoea.^13^ All antibiotic treatments, including those in addition to chronic antibiotic therapy, are documented in the German CF registry, and the reason for therapy can be selected from a predefined list of options in a drop-down menu. Only antibiotic treatments specifically prescribed for pulmonary exacerbations were included in the documentation process and subsequent analysis.

### Statistical analysis

Baseline demographics and clinical characteristics are presented descriptively using mean and standard deviation values or proportions. Because examinations of pwCF occurred on different dates and in different intervals within the registry, consistent time periods were formed for further analysis. Hence, values of percent predicted forced expiratory volume in 1 s (ppFEV_1_), BMI and BMI *z*-score were calculated as the mean for each quarter over the four quarters before and after ETI therapy initiation. Data from the year before ETI therapy initiation are presented to provide an indication of disease progression prior to initiation of treatment. Baseline values were obtained from the last quarter before the initiation of therapy.

Exacerbations were counted dichotomously (yes/no) for every quarter before and after initiation of ETI therapy. The date of ETI initiation was set in the last quarter before documented therapy begin (quarter −1). The first four weeks of therapy were not included in the considered time intervals, so that the first quarter (quarter 1) begins 4 weeks after therapy initiation and ends 17 weeks later. Due to missing values, sweat chloride level was defined as the minimum value in the year before and the year after the start of therapy.

For ppFEV_1_, BMI and BMI *z*-score, differences between individual quarters after therapy initiation were compared with the baseline value using paired t-tests. Gender differences were evaluated using two-sample t-tests. For paired t-tests, the normality assumption for differences was determined using normal Quantile-Quantile-Plots (QQ-Plots). For independent t-tests, normality assumption in each group was assessed using normal QQ-Plots, and homogeneity of variance assumption was tested using F-tests. Further comparisons were performed for pwCF for whom data were available for all four quarters before and after the start of ETI therapy.

McNemar's test was used to analyse how many pwCF experienced a change from ‘exacerbation’ to ‘no exacerbation’, and vice versa. McNemar's test was also used to evaluate changes in categories of ppFEV_1_, BMI and BMI *z*-score categories; the proportion of individuals in the optimal category was compared with that in the other categories. Change in sweat chloride levels from the year before to the year after therapy initiation were analysed using a paired t-test. All p-values calculated in the study were corrected using the Benjamini-Hochberg method. Analyses were conducted using R version 4.2.2.

## Results

### Study population and follow-up

**Fig. 1:**
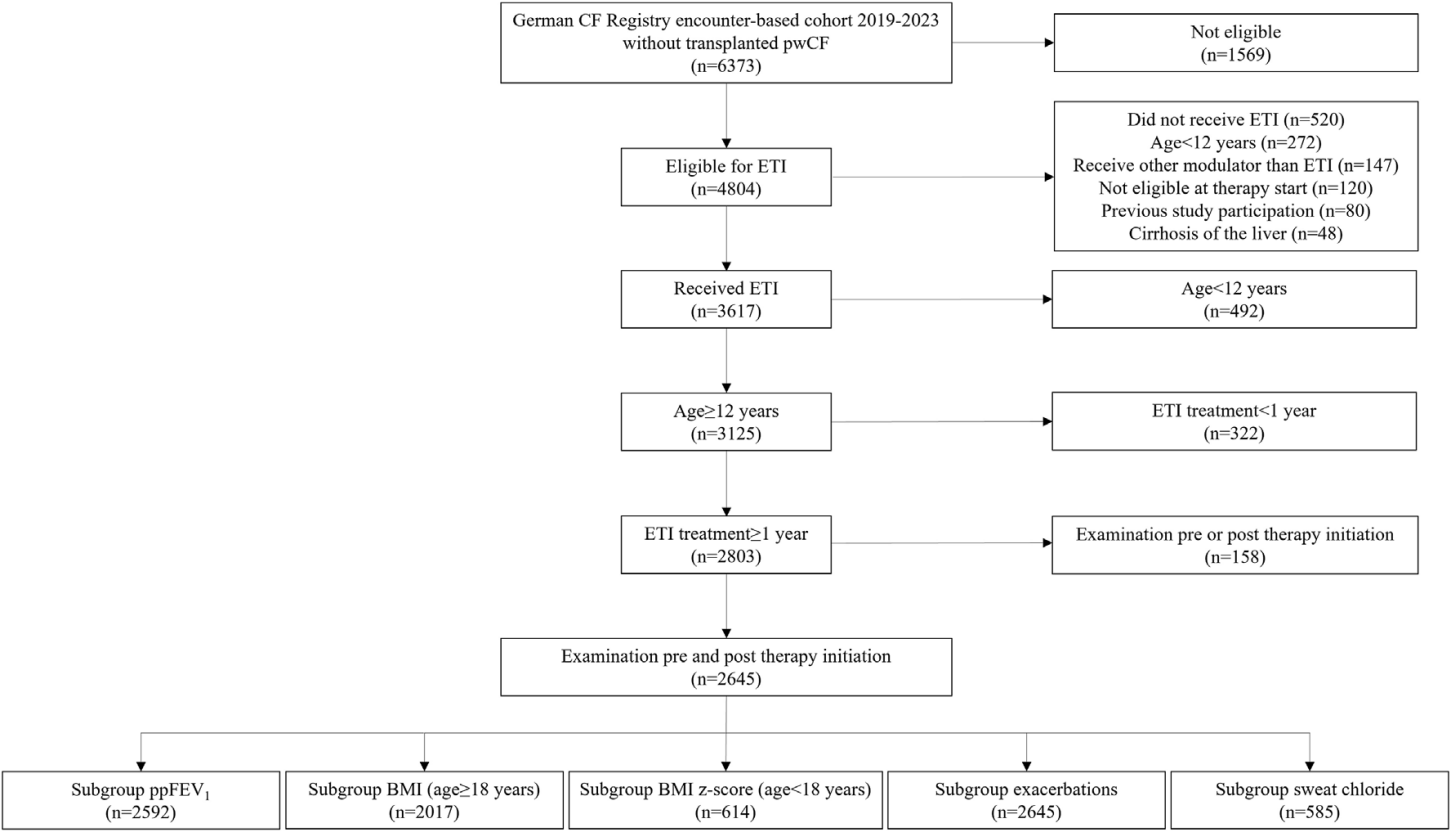
**Participant flow chart**. BMI, body mass index; CF, cystic fibrosis; ETI, elexacaftor/tezacaftor/ivacaftor; ppFEV1, percent predicted forced expiratory volume in 1 s.

Of 6373 pwCF in the registry dataset, 2803 (44%) were aged ≥12 years and received ETI for ≥1 year, and 2645/2803 of these (94%) had examinations before and after therapy initiation (Fig. 1, Table 1). The number of pwCF who had data available for analysis was 2592 for ppFEV_1_, 2017 for BMI in adults, 614 for BMI *z*-score in adolescents, 2645 for exacerbations, and 585 for sweat chloride concentrations (Fig. 1). The majority of pwCF started ETI therapy in the period from September 2020 to September 2021 (Figure S1). There were 371 pwCF who had data for ppFEV_1_ and BMI or BMI z-score available for all four quarters before and after the start of ETI therapy (Table S1).

**Table 1:** Participant demographic and clinical characteristics at baseline.

Variable	N	Value
Age at therapy start, years	2645	28.0 ± 11.5 (12–75)
Adolescents	614	23.2%
Adults	2031	76.8%
Male sex	1363	51.5%
Female sex	1282	48.5%
ppFEV_1_, %	2609	64.7 ± 23.7 (12.7–126.8)
ppFEV_1_ 0–40%	503	19.0%
ppFEV_1_ 40–60%	629	23.8%
ppFEV_1_ 60–80%	700	26.5%
ppFEV_1_ 80–127%	777	29.4%
ppFEV_1_ unknown	36	1.4%
BMI (adults), kg/m^2^	2031	21.3 ± 3.1 (13.6–46.1)
BMI z-score (adolescents)	614	−0.5 ± 1.0 (−5.5, −2.1)
Weight (adults), kg	2031	62.1 ± 12.3 (36.5–127.0)
Weight z-score (adolescents)	614	−0.7 ± 1.1 (−6.1, −2.2)
Mutation		
F508del heterozygous: gating	48	1.8%
F508del heterozygous: minimal function	893	33.8%
F508del heterozygous: residual function	91	3.4%
F508del heterozygous: other	94	3.6%
F508del homozygous	1519	57.4%
Previous CFTR modulator therapy		
No	1439	54.4%
Yes	1206	45.6%

Values are mean ± standard deviation (with or without range), or proportion of patients as a percentage.

BMI, body mass index; CFTR, cystic fibrosis transmembrane conductance regulator; ppFEV_1_, percent predicted forced expiratory volume in 1 s.

### Lung function

**Fig. 2:**
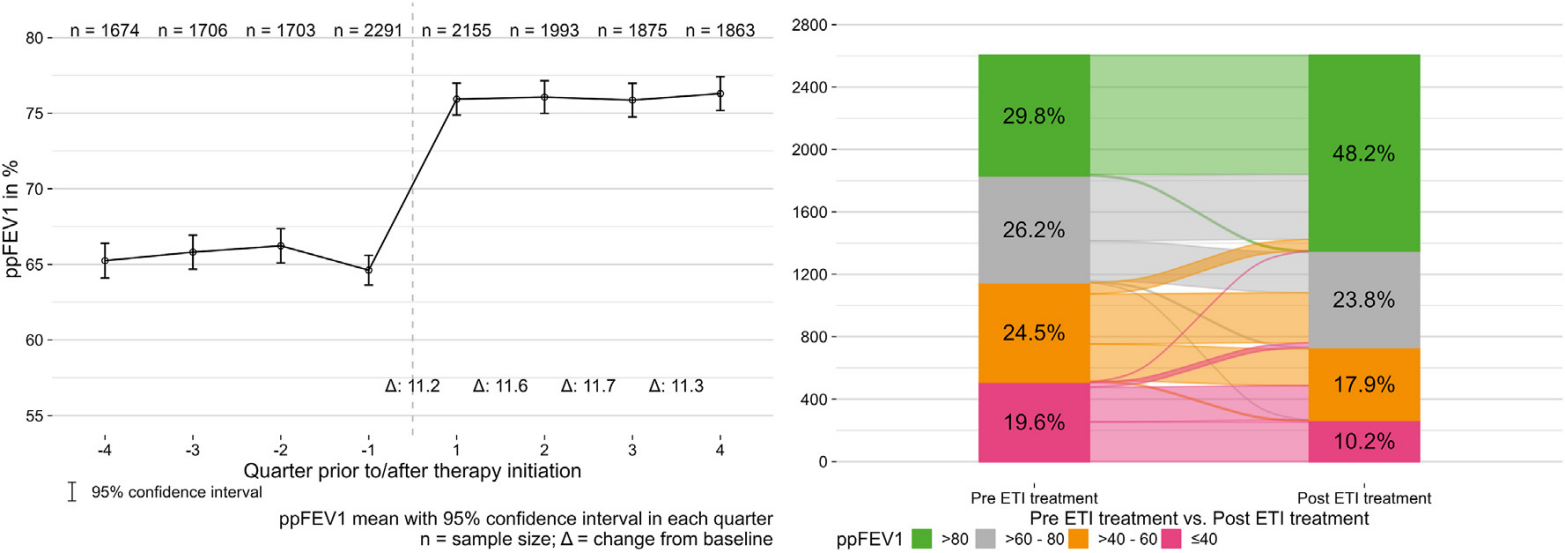
**Change in percent predicted forced expiratory volume in 1 s.** Change over time before and after initiation of elexacaftor/tezacaftor/ivacaftor (ETI) therapy (left panel) and change in severity category from the year before to the year after initiation of elexacaftor/tezacaftor/ivacaftor (ETI) therapy (right panel). ppFEV_1_, percent predicted forced expiratory volume in 1 s.

There was a significant and consistent improvement from baseline in ppFEV_1_ during all four quarters of the year after initiation of ETI therapy (Table 2, Fig. 2). The improvement in ppFEV_1_ during treatment with ETI was significant compared with baseline in the subgroups with and without prior CFTR modulator therapy, but was greater in CFTR-naïve vs. CFTR experienced individuals (+12.6% vs. +9.7%). The proportion of pwCF with ppFEV_1_ in lower categories decreased during ETI therapy while the proportion in higher function categories increased; for example, the proportion with ppFEV_1_ >80% increased from 29.8% before ETI therapy to 48.2% during treatment with ETI (Fig. 2). Findings were consistent when this analysis was repeated with only the 371 pwCF who had ppFEV_1_ values available for every quarter (Figure S2, Table S2). The mean change in ppFEV_1_ from baseline to 12 months after therapy initiation differed by <1% between males (mean 10.9%) and females (11.8%); 95% confidence interval for the between-group difference was −1.98, 0.15 (p = 0.10) (Figure S3).

**Table 2:** Clinical outcomes by study quarter for the overall population, and in subgroups with and without previous cystic fibrosis transmembrane conductance regulator modulator therapy.

	N (change)	Mean ± SD	Change (95% CI)^a^	p-value^b^
**Total population**				
**ppFEV**_**1**_				
Previous 10–12 months	1674 (1494)	65.3 ± 24.1	−0.4 (−0.8, 0.0)	0.0758
Baseline	2291 (−)	64.6 ± 24.0	–	–
ETI therapy 1–3 months	2155 (1937)	75.9 ± 25.0	11.3 (10.8, 11.7)	<0.0001
ETI therapy 4–6 months	1993 (1788)	76.1 ± 24.5	11.6 (11.1, 12.1)	<0.0001
ETI therapy 7–9 months	1875 (1685)	75.9 ± 24.5	11.7 (11.2, 12.3)	<0.0001
ETI therapy 10–12 months	1863 (1667)	76.3 ± 24.6	11.3 (10.8, 11.8)	<0.0001
**BMI (adults), kg/m**^**2**^				
Previous 10–12 months	1329 (1207)	21.2 ± 3.0	0.1 (0.1, 0.2)	<0.0001
Baseline	1826 (−)	21.4 ± 3.2	–	–
ETI therapy 1–3 months	1684 (1541)	22.1 ± 3.2	0.7 (0.7, 0.8)	<0.0001
ETI therapy 4–6 months	1566 (1437)	22.6 ± 3.3	1.2 (1.1, 1.3)	<0.0001
ETI therapy 7–9 months	1507 (1379)	22.7 ± 3.2	1.3 (1.2, 1.4)	<0.0001
ETI therapy 10–12 months	1478 (1349)	22.8 ± 3.2	1.4 (1.3, 1.4)	<0.0001
**BMI z-score (adolescents)**				
Previous 10–12 months	448 (416)	−0.5 ± 1.0	0.0 (0.0, 0.1)	0.0819
Baseline	564 (−)	−0.5 ± 1.1	–	–
ETI therapy 1–3 months	538 (504)	−0.3 ± 0.9	0.3 (0.2, 0.3)	<0.0001
ETI therapy 4–6 months	501 (459)	−0.2 ± 1.0	0.3 (0.3, 0.4)	<0.0001
ETI therapy 7–9 months	460 (428)	−0.2 ± 1.0	0.4 (0.3, 0.4)	<0.0001
ETI therapy 10–12 months	474 (436)	−0.2 ± 1.0	0.3 (0.3, 0.4)	<0.0001
**Exacerbations**				
Previous 10–12 months	2645 (−)	464	−19	0.0326
Baseline	2645 (−)	483	–	–
ETI therapy 1–3 months	2645 (−)	146	−337	<0.0001
ETI therapy 4–6 months	2645 (−)	95	−388	<0.0001
ETI therapy 7–9 months	2645 (−)	107	−376	<0.0001
ETI therapy 10–12 months	2645 (−)	145	−338	<0.0001
**Cftr modulator naïve population**				
**ppFEV**_**1**_				
Previous 10–12 months	854 (753)	68.6 ± 23.9	−0.9 (−1.5, −0.3)	0.0051
Baseline	1249 (−)	66.6 ± 24.1	–	–
ETI therapy 1–3 months	1183 (1064)	79.2 ± 24.8	12.6 (11.9, 13.2)	<0.0001
ETI therapy 4–6 months	1067 (961)	79.5 ± 24.2	13.1 (12.4, 13.8)	<0.0001
ETI therapy 7–9 months	1022 (921)	78.6 ± 24.4	12.9 (12.2, 13.7)	<0.0001
ETI therapy 10–12 months	1007 (903)	79.6 ± 24.3	12.6 (11.9, 13.4)	<0.0001
**BMI (adults), kg/m**^**2**^				
Previous 10–12 months	629 (567)	21.4 ± 3.0	0.1 (−0.0, 0.2)	0.1007
Baseline	948 (−)	21.6 ± 3.2	–	–
ETI therapy 1–3 months	880 (807)	22.3 ± 3.2	0.7 (0.7, 0.8)	<0.0001
ETI therapy 4–6 months	785 (724)	22.7 ± 3.3	1.2 (1.1, 1.3)	<0.0001
ETI therapy 7–9 months	779 (715)	22.8 ± 3.2	1.3 (1.2, 1.4)	<0.0001
ETI therapy 10–12 months	757 (690)	22.9 ± 3.2	1.3 (1.2, 1.5)	<0.0001
**BMI z-score (adolescents)**				
Previous 10–12 months	272 (252)	−0.6 ± 1.0	0.0 (−0.0, 0,1)	0.2306
Baseline	352 (−)	−0.6 ± 1.1	–	–
ETI therapy 1–3 months	335 (312)	−0.3 ± 1.0	0.3 (0.3, 0.3)	<0.0001
ETI therapy 4–6 months	316 (292)	−0.3 ± 1.0	0.4 (0.3, 0.4)	<0.0001
ETI therapy 7–9 months	287 (266)	−0.2 ± 1.0	0.4 (0.4, 0.5)	<0.0001
ETI therapy 10–12 months	297 (274)	−0.2 ± 1.0	0.4 (0.4, 0.5)	<0.0001
**Exacerbations**				
Previous 10–12 months	1439 (−)	229	−48	0.0002
Baseline	1439 (−)	277	–	–
ETI therapy 1–3 months	1439 (−)	79	−198	<0.0001
ETI therapy 4–6 months	1439 (−)	50	−227	<0.0001
ETI therapy 7–9 months	1439 (−)	54	−223	<0.0001
ETI therapy 10–12 months	1439 (−)	78	−199	<0.0001
**Previous cftr modulator use population**				
**ppFEV**_**1**_				
Previous 10–12 months	820 (741)	61.8 ± 23.7	0.1 (−0.5, 0.7)	0.7279
Baseline	1042 (−)	62.2 ± 23.7	–	–
ETI therapy 1–3 months	972 (873)	72.0 ± 24.7	9.6 (9.0, 10.3)	<0.0001
ETI therapy 4–6 months	926 (827)	72.1 ± 24.2	9.9 (9.2, 10.5)	<0.0001
ETI therapy 7–9 months	853 (764)	72.6 ± 24.3	10.3 (9.6, 11.0)	<0.0001
ETI therapy 10–12 months	856 (764)	72.4 ± 24.4	9.7 (9.0, 10.5)	<0.0001
**BMI (adults), kg/m**^**2**^				
Previous 10–12 months	700 (640)	21.1 ± 3.0	0.2 (0.1, 0.3)	<0.0001
Baseline	878 (−)	21.3 ± 3.2	–	–
ETI therapy 1–3 months	804 (734)	22.0 ± 3.2	0.8 (0.7, 0.8)	<0.0001
ETI therapy 4–6 months	781 (713)	22.4 ± 3.3	1.2 (1.1, 1.3)	<0.0001
ETI therapy 7–9 months	728 (664)	22.5 ± 3.2	1.3 (1.2, 1.5)	<0.0001
ETI therapy 10–12 months	721 (659)	22.6 ± 3.3	1.4 (1.2, 1.5)	<0.0001
**BMI z-score (adolescents)**				
Previous 10–12 months	176 (164)	−0.5 ± 0.9	0.0 (−0.0, 0.11)	0.1989
Baseline	212 (−)	−0.4 ± 0.9	–	–
ETI therapy 1–3 months	203 (192)	−0.2 ± 0.9	0.2 (0.1, 0.3)	<0.0001
ETI therapy 4–6 months	185 (167)	−0.2 ± 0.9	0.3 (0.2, 0.4)	<0.0001
ETI therapy 7–9 months	173 (162)	−0.2 ± 0.9	0.2 (0.1, 0.3)	<0.0001
ETI therapy 10–12 months	177 (162)	−0.2 ± 0.9	0.2 (0.1, 0.3)	<0.0001
**Exacerbations**				
Previous 10–12 months	1206 (−)	235	29	0.0002
Baseline	1206 (−)	206	–	–
ETI therapy 1–3 months	1206 (−)	67	−139	<0.0001
ETI therapy 4–6 months	1206 (−)	45	−161	<0.0001
ETI therapy 7–9 months	1206 (−)	53	−153	<0.0001
ETI therapy 10–12 months	1206 (−)	67	−139	<0.0001

BMI, body mass index; CI, confidence interval; ETI, elexacaftor/tezacaftor/ivacaftor; ppFEV_1_, percent predicted forced expiratory volume in 1 s; SD, standard deviation.

a Change is calculated as mean minus baseline mean except for the ‘Previous 10–12 months’ rows where change is calculated as baseline minus the mean for the 10–12 months before starting ETI.

b p-values were calculated using paired t-test for ppFEV_1_, BMI and BMI z-score, and McNemar's test for exacerbations. All p-values and were corrected using the Benjamini-Hochberg method.

### BMI

**Fig. 3:**
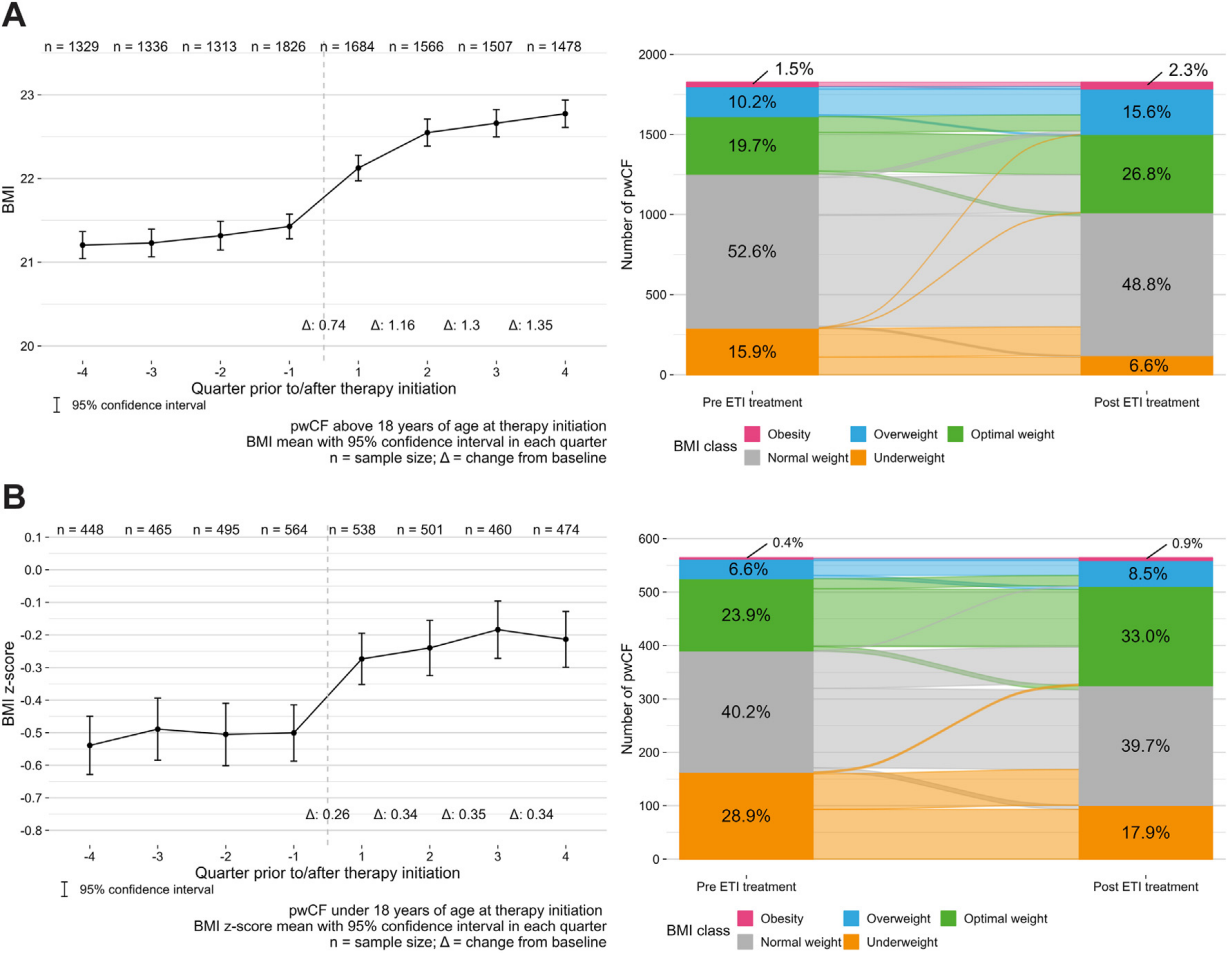
**Change in body mass index**. Changes over time before and after initiation of elexacaftor/tezacaftor/ivacaftor (ETI) therapy (left panels) and changes in severity category from the year before to the year after initiation of ETI therapy (right panels) for adults (**A**) and adolescents (**B**). BMI, body mass index; pwCF, people with cystic fibrosis.

In adult pwCF, BMI increased significantly from a mean of 21.2 kg/m^2^ from one quarter prior to ETI to 22.8 kg/m^2^ after one year of treatment (Fig. 3A, Table 2). Results were consistent in subgroups with and without previous CFTR modulator therapy (Table 2), and after analysing only individuals with BMI values in all four quarters (Table S2, Figure S4). The proportion of underweight individuals decreased during ETI therapy, while the proportion of overweight or obese individuals increased (Fig. 3A). There was almost no difference in the change in BMI from baseline to 12 months between males (mean 1.35 kg/m^2^) and females (mean 1.34 kg/m^2^); between-group difference 0.01 (95% confidence interval −0.15, 0.18; p = 0.87) (Figure S5). A total of 2313 pwCF provided information on supplemental feeding both before and after ETI therapy initiation. Prior to starting ETI therapy, 693/2313 (30.0%) pwCF indicated the use of supplemental feeding, and 559/2313 (24.2%) reported use of supplemental feeding after one year of ETI therapy (Table S3).

### BMI *z*-score

In adolescent pwCF, the BMI *z*-score increased significantly from one quarter prior to 1 year after ETI therapy initiation (Fig. 3B, Table 2). Again, findings were consistent when only those with BMI *z*-score values available in all four quarters before and after ETI therapy initiation were analysed (Table S2, Figure S6). As for adults, the proportion of underweight adolescent individuals decreased during ETI therapy, while the proportion of overweight or obese individuals increased (Fig. 3B). There was very little difference in mean BMI *z*-score between males (0.36) and females (0.32); between-group difference 0.04 (95% confidence interval −0.08, 0.15; (p = 0.52) (Figure S7).

### Sweat chloride

Overall, mean sweat chloride concentration decreased from 97.0 ± 20.4 mmol/L in the year prior to ETI start to 46.1 ± 20.4 mmol/L within one year of treatment with ETI (change of −50.9 mmol/L; 95% confidence interval −52.6, −49.3); p < 0.0001); reductions were similar in the subgroups with or without prior CFTR modulator therapy (Figure S8). Reductions in sweat chloride concentration were greater in females (from 95.2 to 40.9 mmol/L) than in males (from 98.7 to 50.8 mmol/L); change of −54.3 vs. −47.9 mmol/L, respectively; p < 0.0001) (Figure S9).

### Exacerbations

**Fig. 4:**
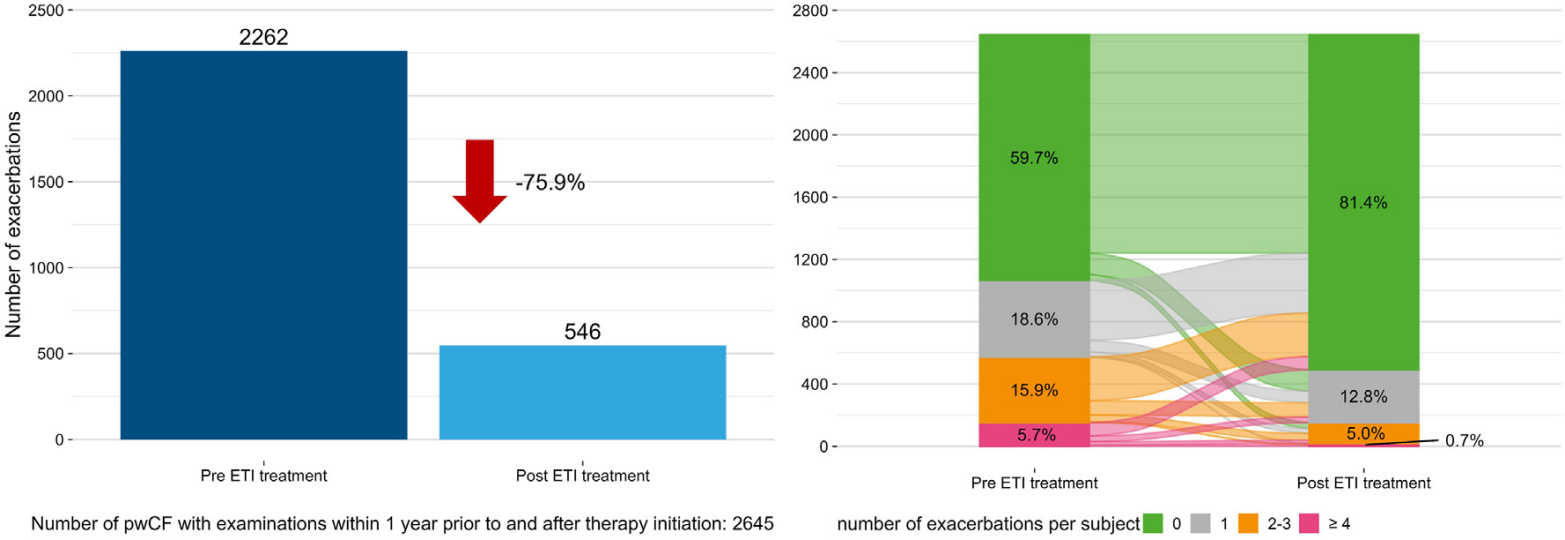
**Change in number of exacerbations**. Change in total number of exacerbations in the year before and the year after initiation of elexacaftor/tezacaftor/ivacaftor (ETI) therapy (left panel) and change in exacerbation severity category in the year before vs. the year after initiation of ETI therapy (right panel). pwCF, people with cystic fibrosis.

There were 2262 exacerbations in the year before starting ETI and 546 exacerbations in the year after therapy initiation (−75.9%; p ≤ 0.0001) (Fig. 4); the reduction in the number of pwCF with exacerbations was slightly smaller in the subgroup with examinations in both the year before and the year after therapy (−62.2%) (Figure S10). Details of the number of pwCF who experienced exacerbations in each quarter of the years before and after the initiation of ETI therapy are shown in Figure S11. In addition, the proportion of pwCF without an exacerbation increased in the year after ETI therapy, while the proportion with ≥4 exacerbations decreased (Fig. 4).

## Discussion

The findings of this real-world study showed statistically significant improvements in clinical outcomes 12 months after initiation of ETI, including improved ppFEV_1_, increased BMI, decreased sweat chloride levels, and a reduction in the number of pulmonary exacerbations. These changes in lung function, nutritional status, pulmonary exacerbation frequency and sweat chloride concentrations were consistent with data from randomised clinical trials of ETI in pwCF.^7^, ^8^, ^9^, ^10^, ^11^^,^^14^ This comparability between data from clinical trials and that obtained in the current real-world data analysis from the German CF registry affirms the quality of the data acquired from the German CF registry and reinforces the value of real-world evidence.

CF registries have contributed significantly to improving understanding of the disease and its impact on health outcomes. With the emergence of big data, these registries can now be linked to various data sources to evaluate the effectiveness of different treatments in real-world settings. Given the limited feasibility of traditional clinical trial methods for rare diseases such as CF, real-world evidence could play a pivotal role in supporting the approval of new treatments.^15^, ^16^, ^17^, ^18^, ^19^, ^20^

Improved lung function was seen in all pwCF in the current analysis, but improvements were greater in CFTR modulator-naïve individuals, consistent with the findings of recent post authorization trials.^21^ In CFTR modulator-naïve pwCF there was a decline in ppFEV_1_ in the year before ETI therapy, and this was followed by an increase in the first quarter after starting ETI, similar to interim results from the open-label phase 3 study for ETI.^14^ Furthermore, we identified a shift towards better ppFEV_1_ severity classification, meaning that fewer individuals had a ppFEV_1_ of ≤40% after treatment with ETI. For pwCF who have advanced lung disease and a ppFEV_1_ of ≤40%, experiencing an improvement in their condition and transitioning to the next ppFEV_1_ range of 40–60% is a significant outcome. In addition, our findings confirm previous data showing a decrease in the proportion of pwCF with advanced lung disease.^22^ Thus, ETI has a positive effect not only in individuals with mild-to-moderate CF but also in those with advanced pulmonary disease, including candidates for lung transplantation.^4^^,^^18^^,^^22^

The improvement in BMI after 12 months of ETI treatment in adult pwCF in the current analysis was +1.4 kg/m^2^, which is in a similar range to that reported in previous studies (+1.2 kg/m^2^ after 6 months in the PROMISE study^21^ and +1.5 kg/m^2^ after 12 months in another study^23^). Furthermore, the trends we observed in transitions from underweight to normal weight or optimal weight to overweight and obesity were similar to those reported previously.^23^ To the best of our knowledge, the current study is the first to replicate these BMI findings in adolescents, who showed changes in BMI *z*-score classes after ETI initiation, with a decreasing proportion of underweight individuals and slight increases in the proportion who were overweight or obese. After ETI therapy in our study, a considerable number of individuals with CF who had previously maintained a normal weight were categorised as overweight or obese. Furthermore, despite achieving a healthy weight, a notable proportion of these individuals were still being prescribed supplemental feedings. This suggests that continued high-calorie supplemental feeding, in addition to other factors such as ETI, may have contributed to their increased weight.^23^^,^^24^ Thus, nutrition issues should remain a focus of management for individuals with CF, even after reaching a healthy weight.^23^, ^24^, ^25^ As the prevalence of overweight and obesity in people with CF, including adolescents, continues to rise, it may be necessary to revisit nutritional guidelines, particularly for indication for high-energy diets, in order to better manage the risks associated with weight gain and obesity-related diseases.^25^ Taken together, our data highlight the need for careful monitoring of weight status and co-morbidities associated with obesity, along with timely consideration of alternative dietary strategies in the management of CF.^24^

Obesity is known to increase the risk of comorbidities such as hypertension, obstructive sleep apnoea, type 2 diabetes, and cancer, and may also negatively impact lung transplant outcomes in people with CF. In addition, it has also be shown that an increase in BMI towards the overweight or obesity categories is associated with elevations in blood pressure.^11^ Together, these data suggest that cardiovascular risk may increase in pwCF treated with ETI, highlighting a need for the introduction of regular cardiovascular and cardiometabolic screening.

In the first year after starting treatment with ETI, the total number of documented pulmonary exacerbations requiring antibiotic treatment was reduced by 75.9%. In a phase III study in pwCF and a single copy of F508del, the number of exacerbations was reduced by 62.2% after 24 weeks of treatment with ETI.^9^ Another study in pwCF and advanced lung disease showed that treatment with ETI for 48 weeks reduced the rate of pulmonary exacerbations (also defined based on change in use of any antibiotic) by 97%.^26^ In the latter study, the authors speculated that limitations on contact outside the household during the COVID 19 pandemic may have contributed to the near elimination of pulmonary exacerbations seen. The majority of the current study also took place during the COVID-19 pandemic due to the timing of approval of ETI in Europe. Thus, although there is no doubt that treatment with ETI reduces the number of pulmonary exacerbations, we cannot rule out the possibility that measures such as self-isolation and wearing masks during the COVID-19 pandemic could also have contributed to the reduction in the number of exacerbations seen. A more detailed analysis of exacerbations over each year since the start of the COVID-19 pandemic is planned and might provide better insight. Regardless of the contributing factors, a reduction in the number of exacerbations could contribute to better long-term outcomes for pwCF (e.g., survival) because exacerbations are a risk factor for faster decline in FEV_1_.^27^

As a clinical marker of CFTR function, the sweat chloride concentration is related to disease severity and is predictive of mortality, lung function and BMI.^28^ The absolute change in sweat chloride concentration from 97.0 mmol/L in the year before ETI to 46.1 mmol/L in the year after initiation of ETI in our cohort is greater than that in studies including only F508del homozygous pwCF.^8^^,^^11^ The absolute mean change of 50.9 mmol/L in the current study was also greater than that in the PROMISE study (41.7 mmol/L after 6 months).^21^ As in the PROMISE study,^21^ we found a significant difference between males and females regarding the change in sweat chloride concentration, but not for BMI and ppFEV_1_ after initiation of ETI.

Since the licensing of ETI in the US in November 2019 and in Europe in August 2020, the current study represents the largest cohort of pwCF receiving ETI treatment from Europe and the study includes a follow-up period of 12 months. In comparison, the recent PROMISE study reported on a smaller cohort (487 pwCF) and a shorter follow-up (six months).^21^ Despite this important strength regarding sample size and follow-up duration, a number of limitations need to be considered. Real-world evidence (RWE) studies may have selection bias because the pwCF included might not be representative of the overall population due to the impact of specific factors on inclusion, including medical conditions or treatment history. Furthermore, the external validity of the current study may be limited due to the specific nature of the study population (i.e., all pwCF were from Germany). When evaluating RWE studies such as ours, two important factors should be considered: data quality and confounding factors. Real-world studies typically rely on electronic health records or administrative claims data, which may lack relevant information or contain errors. Additionally, unmeasured or uncontrolled confounding factors, such as lifestyle or environmental factors, can influence the results of RWE studies.^15^ The German CF Registry does not currently document side effects related to treatment with CFTR modulators. This represents a limitation in our data, especially in the context of growing concerns regarding intolerance of CFTR modulator therapy. Finally, the pre-post design of the analysis and the lack of a control group has some inherent limitations. For example, differences between pre- and post-values can be affected by factors other than the treatment of interest (e.g., biological changes), and regression to the mean can lead to misinterpretation of natural variation in repeated data as a real change.

In conclusion, these findings provide important insights into the real-world impact of ETI on pwCF in Germany. The data show that ETI therapy provides significant clinical benefits in a heterogenous group of pwCF. In addition, the BMI data highlight the importance of carefully monitoring weight status and performing cardiovascular screening to help mitigate any potential risks of body weight changes after initiation of ETI.

## Contributors

Study conception: SS, LN, MB.

Data acquisition and analysis: all authors.

Data interpretation: all authors.

Writing the original manuscript: SS, SD, LN, SN, SaS.

Revising the work for important intellectual content: all authors.

All authors contributed to the article and approved the submitted version.

## Data sharing statement

Data that underline the results reported in this article and the respective individual participant data will not be shared.

## Declaration of interests

SS received personal fees or grants from Galapagos, Proteostasis Therapeutics, Celtaxsys, Vertex Pharmaceuticals, Boehringer Ingelheim, Corbus Pharmaceuticals, Insmed Germany GmbH and Ionis Pharmaceuticals outside the submitted work. SD participated in the Advance program, financially supported by Vertex Pharmaceuticals. MW received personal fees from Vertex Pharmaceuticals, Chiesi, CSL Behring, and Grifols outside of the submitted work. CSm received personal fees from Vertex Pharmaceuticals outside the submitted work. FS has no conflict of interest. FB has no conflict of interest. MB receives payments from Mukoviszidose Institut gGmbH. AMD received personal fees from Vertex outside of the submitted work and institutional payments from Vertex for the conduct of clinical studies. HE received personal fees Vertex Pharmaceuticals and Insmed Germany GmbH outside the submitted work. CS received personal fees or grants from Chiesi, GlaxoSmithKline, Boehringer Ingelheim, Vertex Pharmaceuticals and GILEAD outside the submitted work. OE received personal fees or grants from Boerhringer Ingelheim, Chiesi, Corbus Pharmaceuticals, GILEAD, Novartis, Vertex Pharmaceuticals outside the submitted work. MK has no conflict of interest. SaS receives payments for statistical analysis of data that were made to STAT-UP Statistical Consulting & Data Science GmbH. SN received institutional payments from Vertex for the conduct of clinical studies. LN received institutional payments from The German Center of Lung research and Vertex Pharmaceuticals for the conduct of clinical studies, was the medical lead of the German CF Registry and the Pharmacovigilance Study manager of the European Cystic Fibrosis Society Patient Registry and received Medial Writing support from Articulate Science.

## Appendix A Supplementary data

Supplementary Tables Figures

Appendix List

## References

[1] Bell S.C., Mall M.A., Gutierrez H. (2020). The future of cystic fibrosis care: a global perspective. Lancet Respir Med.

[2] Guo J., Garratt A., Hill A. (2022). Worldwide rates of diagnosis and effective treatment for cystic fibrosis. J Cyst Fibros.

[3] Naehrlich L., Burkhart M., Basler C. (2021).

[4] Shteinberg M., Haq I.J., Polineni D., Davies J.C. (2021). Cystic fibrosis. Lancet.

[5] Birket S.E., Chu K.K., Liu L. (2014). A functional anatomic defect of the cystic fibrosis airway. Am J Respir Crit Care Med.

[6] Ratjen F., Bell S.C., Rowe S.M., Goss C.H., Quittner A.L., Bush A. (2015). Cystic fibrosis. Nat Rev Dis Primers.

[7] Barry P.J., Mall M.A., Álvarez A. (2021). Triple therapy for cystic fibrosis Phe508del-gating and -residual function genotypes. N Engl J Med.

[8] Heijerman H.G.M., McKone E.F., Downey D.G. (2019). Efficacy and safety of the elexacaftor plus tezacaftor plus ivacaftor combination regimen in people with cystic fibrosis homozygous for the F508del mutation: a double-blind, randomised, phase 3 trial. Lancet.

[9] Middleton P.G., Mall M.A., Dřevínek P. (2019). Elexacaftor-Tezacaftor-Ivacaftor for Cystic Fibrosis with a Single Phe508del Allele. N Engl J Med.

[10] Ramsey B.W., Davies J., McElvaney N.G. (2011). A CFTR potentiator in patients with cystic fibrosis and the G551D mutation. N Engl J Med.

[11] Sutharsan S., McKone E.F., Downey D.G. (2022). Efficacy and safety of elexacaftor plus tezacaftor plus ivacaftor versus tezacaftor plus ivacaftor in people with cystic fibrosis homozygous for F508del-CFTR: a 24-week, multicentre, randomised, double-blind, active-controlled, phase 3b trial. Lancet Respir Med.

[12] Neuhauser H., Schienkiewitz A., Rosario A.S., Dortschy R., Kurth B. (2013). Referenzperzentile für anthropometrische Maßzahlen und Blutdruck aus der Studie zur Gesundheit von Kindern und Jugendlichen in Deutschland (KiGGS). https://www.rki.de/DE/Content/Gesundheitsmonitoring/Gesundheitsberichterstattung/GBEDownloadsB/KiGGS_Referenzperzentile.pdf.

[13] Bilton D., Canny G., Conway S. (2011). Pulmonary exacerbation: towards a definition for use in clinical trials. Report from the EuroCareCF Working Group on outcome parameters in clinical trials. J Cyst Fibros.

[14] Griese M., Costa S., Linnemann R.W. (2021). Safety and Efficacy of Elexacaftor/Tezacaftor/Ivacaftor for 24 Weeks or Longer in People with Cystic Fibrosis and One or More F508del Alleles: interim Results of an Open-Label Phase 3 Clinical Trial. Am J Respir Crit Care Med.

[15] Dang A. (2023). Real-world evidence: a primer. Pharmaceut Med.

[16] Dasenbrook E.C., Sawicki G.S. (2018). Cystic fibrosis patient registries: a valuable source for clinical research. J Cyst Fibros.

[17] Maruszczyk K., Aiyegbusi O.L., Torlinska B., Collis P., Keeley T., Calvert M.J. (2022). Systematic review of guidance for the collection and use of patient-reported outcomes in real-world evidence generation to support regulation, reimbursement and health policy. J Patient Rep Outcomes.

[18] Ringshausen F.C., Sauer-Heilborn A., Büttner T. (2023). Lung transplantation for end-stage cystic fibrosis before and after the availability of elexacaftor-tezacaftor-ivacaftor, Germany, 2012-2021. Eur Respir J.

[19] Schad F., Thronicke A. (2022). Real-world evidence-current developments and perspectives. Int J Environ Res Public Health.

[20] Wu J., Wang C., Toh S., Pisa F.E., Bauer L. (2020). Use of real-world evidence in regulatory decisions for rare diseases in the United States-Current status and future directions. Pharmacoepidemiol Drug Saf.

[21] Nichols D.P., Paynter A.C., Heltshe S.L. (2022). Clinical effectiveness of elexacaftor/tezacaftor/ivacaftor in people with cystic fibrosis: a clinical trial. Am J Respir Crit Care Med.

[22] Burgel P.R., Durieu I., Chiron R. (2021). Rapid improvement after starting elexacaftor-tezacaftor-ivacaftor in patients with cystic fibrosis and advanced pulmonary disease. Am J Respir Crit Care Med.

[23] Petersen M.C., Begnel L., Wallendorf M., Litvin M. (2022). Effect of elexacaftor-tezacaftor-ivacaftor on body weight and metabolic parameters in adults with cystic fibrosis. J Cyst Fibros.

[24] Gabel M.E., Fox C.K., Grimes R.A. (2022). Overweight and cystic fibrosis: an unexpected challenge. Pediatr Pulmonol.

[25] Welter J.J., Lennox A.T., Krishnan S. (2022). The relationship between weight and pulmonary outcomes in overweight and obese people with cystic fibrosis: a retrospective observational study. Health Sci Rep.

[26] Carnovale V., Iacotucci P., Terlizzi V. (2022). Elexacaftor/Tezacaftor/Ivacaftor in Patients with Cystic Fibrosis Homozygous for the F508del Mutation and Advanced Lung Disease: a 48-Week Observational Study. J Clin Med.

[27] de Boer K., Vandemheen K.L., Tullis E. (2011). Exacerbation frequency and clinical outcomes in adult patients with cystic fibrosis. Thorax.

[28] Accurso F.J., Van Goor F., Zha J. (2014). Sweat chloride as a biomarker of CFTR activity: proof of concept and ivacaftor clinical trial data. J Cyst Fibros.

