# Supplementary material for: Impact of elexacaftor/tezacaftor/ivacaftor on lung function, nutritional status, pulmonary exacerbation frequency and sweat chloride in people with cystic fibrosis: real-world evidence from the German CF Registry
Source: Lancet Reg Health Eur. 2023 Jul 28;32:100690. doi: 10.1016/j.lanepe.2023.100690 (PMC10405057; doi:10.1016/j.lanepe.2023.100690)

**ONLINE SUPPLEMENT**

**Impact of elexacaftor/tezacaftor/ivacaftor on lung function, nutritional status, pulmonary exacerbation frequency and sweat chloride in people with cystic fibrosis: real-world evidence from the German CF Registry**

Sivagurunathan Sutharsan^#1^, Stefanie Dillenhoefer^#2^, Matthias Welsner^1^, Florian Stehling^3^, Folke Brinkmann^4^, Manuel Burkhart^5^, Helmut Ellemunter^6^, Anna-Maria Dittrich^7^, Christina Smaczny^8^, Olaf Eickmeier^9^, Matthias Kappler^10^, Carsten Schwarz^11^, Sarah Sieber^12^, Susanne Naehrig^#13^, Lutz Naehrlich^#14, #15^, on behalf of the German CF Registry of the Mukoviszidose e.V. and participating CF sites

^1^Department of Pulmonary Medicine, University Hospital Essen - Ruhrlandklinik, Adult Cystic Fibrosis Center, University of Duisburg-Essen, Essen, Germany

^2^Department of Pediatric Pulmonology, Cystic Fibrosis Center, University Children’s Hospital of Ruhr University Bochum at St. Josef-Hospital, Bochum, Germany

^3^Pediatric Pulmonology and Sleep Medicine, Children's University Hospital Essen, University of Duisburg-Essen, Essen, Germany

^4^Department of Pediatric Pneumology & Allergology, The University of Lübeck, University Medical Center Schleswig-Holstein, Campus Centrum Lübeck, Member of Airway Research Center North (ARCN) of the German Center of Lung Research (DZL), Lübeck, Germany

^5^Mukoviszidose Institut gGmbH (MI), Bonn, Germany

^6^Medical University of Innsbruck, Cystic Fibrosis Centre Innsbruck, Innsbruck, Austria

^7^Department of Paediatric Pneumology, Allergology and Neonatology, Hannover Medical School, Hannover, Germany; Biomedical Research in Endstage and Obstructive Lung Disease Hannover (BREATH), Member of the German Center for Lung Research (DZL), Germany

^8^University Hospital Frankfurt/Main, Goethe University, Pneumology and Allergology, Christiane Herzog CF Center Frankfurt/Main, Frankfurt/Main, Germany

^9^Pediatric Allergology, Pulmonology & Cystic Fibrosis; Christiane Herzog CF Center- Frankfurt a.M., University Hospital Frankfurt a.M., Germany

^10^Department of Pediatrics, Dr. von Hauner Children’s Hospital, University Hospital, LMU Munich, Germany

^11^HMU-Health and Medical University Potsdam, Clinic Westbrandenburg, Division Cystic Fibrosis, Potsdam, Germany

^12^STAT-UP Statistical Consulting & Data Science GmbH, Munich, Germany

^13^Department of Internal Medicine V, Cystic Fibrosis Center for Adults, University Hospital, Ludwig Maximilian University (LMU) Munich, Germany

^14^Department of Pediatrics, Justus-Liebig-University Giessen, Giessen, Germany

^15^Universities of Giessen and Marburg Lung Center (UGMLC), German Center for Lung Research (DZL), Giessen, Germany

**Table S1.** Demographic and clinical characteristics at baseline for participants with data available for all four quarters both before and after initiation of elecacaftor/tezacaftor/ivacaftor therapy

| **Variable** | **N** | **Value** |
| --- | --- | --- |
| Age at therapy start, years | 371 | 25·7±10·9 (12–75) |
| Adolescents | 112 | 30·2% |
| Adults | 259 | 69·8% |
| Male sex | 184 | 49·6% |
| Female sex | 187 | 50·4% |
| ppFEV_1_, % | 371 | 64·6±23·0 (16·3–121·2) |
| ppFEV_1_ 0–40% | 67 | 18·1% |
| ppFEV_1_ 40–60% | 93 | 25·1% |
| ppFEV_1_ 60–80% | 106 | 28·6% |
| ppFEV_1_ 80–127% | 105 | 28·3% |
| BMI (adults), kg/m^2^ | 259 | 21·1±3·0 (14·1–31·6) |
| BMI z-score (adolescents) | 112 | –0·6±0·9 (–3·1–1·6) |
| Weight (adults), kg | 259 | 61·2±12.5 (36·5–119·5) |
| Weight z-score (adolescents) | 112 | –0·8±1·0 (–3·4–1·8) |
| Mutation |  |  |
| F508del heterozygous: gating | 4 | 1·1% |
| F508del heterozygous: minimal function | 133 | 35·8% |
| F508del heterozygous: residual function | 11 | 3·0% |
| F508del heterozygous: other | 8 | 2·2% |
| F508del homozygous | 215 | 58·0% |
| Previous CFTR modulator therapy |  |  |
| No | 185 | 49·9% |
| Yes | 186 | 50·1% |

Values are mean ± standard deviation (with or without range), or proportion of patients as a percentage.

BMI, body mass index; CFTR, cystic fibrosis transmembrane conductance regulator; ppFEV_1_, percent predicted forced expiratory volume in 1 second.

**Table S2.** Clinical outcomes by study quarter for the subset of participants with data available for every quarter, both overall and in subgroups with and without previous cystic fibrosis transmembrane conductance regulator modulator therapy

|  |  |  | **Mean ± SD** | **Change (95% CI)*** | **p-value**** |
| --- | --- | --- | --- | --- | --- |
| **TOTAL POPULATION (n=371)** | | |  |  |  |
| **PpFEV_1_** | | |  |  |  |
| Previous 10–12 months | | | 64·9±23·4 | –0·5 (–1·5, 0·3) | 0·2663 |
| *Baseline* | | | 64·4±23·4 | - | - |
| ETI therapy 1–3 months | | | 76·0±24·3 | 11·6 (10·6, 12·6) | <0·0001 |
| ETI therapy 4–6 months | | | 76·6±24·4 | 12·2 (11·2, 13·3) | <0·0001 |
| ETI therapy 7–9 months | | | 76·7±24·2 | 12·4 (11·3, 13·4) | <0·0001 |
| ETI therapy 10–12 months | | | 76·3±24·0 | 11·9 (10·9, 13·1) | <0·0001 |
| **BMI (adults), kg/m^2^ (n=259)** | | |  |  |  |
| Previous 10–12 months | | | 21·0±3·0 | 0·2 (0·1, 0·4) | 0·0017 |
| *Baseline* | | | 21·2±3·1 | - | - |
| ETI therapy 1–3 months | | | 22·0±3·2 | 0·9 (0·7, 1·0) | <0·0001 |
| ETI therapy 4–6 months | | | 22·5±3·1 | 1·3 (1·1, 1·4) | <0·0001 |
| ETI therapy 7–9 months | | | 22·6±3·1 | 1·4 (1·2, 1·6) | <0·0001 |
| ETI therapy 10–12 months | | | 22·7±3·2 | 1·5 (1·3, 1·7) | <0·0001 |
| **BMI z-score (adolescents) (n=112)** | | |  |  |  |
| Previous 10–12 months | | | –0·7±0·9 | 0·1 (–0·0, 0·2) | 0·0273 |
| *Baseline* | | | –0·6±0·9 | - | - |
| ETI therapy 1–3 months | | | –0·3±0·8 | 0·3 (0·2, 0·4) | <0·0001 |
| ETI therapy 4–6 months | | | –0·2±0·9 | 0·4 (0·3, 0·4) | <0·0001 |
| ETI therapy 7–9 months | | | –0·2±0·9 | 0·4 (0·3, 0·5) | <0·0001 |
| ETI therapy 10–12 months | | | –0·2±0·9 | 0·3 (0·2, 0·5) | <0·0001 |
| **CFTR MODULATOR NAÏVE POPULATION (n=185)** | | | |  |  |
| **ppFEV_1_** | | |  |  |  |
| Previous 10–12 months | | | 68·2±23·3 | –1·6 (–2·8, –0·3) | 0·0145 |
| *Baseline* | | | 66·6±22·8 | - | - |
| ETI therapy 1–3 months | | | 79·9±23·4 | 13·3 (11·8, 14·8) | <0·0001 |
| ETI therapy 4–6 months | | | 80·7±23·7 | 14·2 (12·6, 15·8) | <0·0001 |
| ETI therapy 7–9 months | | | 80·4±23·3 | 13·8 (12·2, 15·4) | <0·0001 |
| ETI therapy 10–12 months | | | 80·0±23·1 | 13·4 (11·7, 15·1) | <0·0001 |
| **BMI (adults), kg/m^2^** | | |  |  |  |
| Previous 10–12 months | | | 21·1±2·9 | 0·1 (–0·1, 0·4) | 0·2306 |
| *Baseline* | | | 21·2±3·1 | - | - |
| ETI therapy 1–3 months | | | 22·0±3·1 | 0·8 (0·6, 1·0) | <0·0001 |
| ETI therapy 4–6 months | | | 22·6±3·1 | 1·4 (1·2, 1·7) | <0·0001 |
| ETI therapy 7–9 months | | | 22·8±3·0 | 1·6 (1·3, 1·9) | <0·0001 |
| ETI therapy 10–12 months | | | 22·8±3·1 | 1·6 (1·4, 2·0) | <0·0001 |
| **BMI z-score (adolescents)** | | |  |  |  |
| Previous 10–12 months | | | –0·6±0·9 | 0·1 (0·0, 0·2) | 0·1785 |
| *Baseline* | | | –0·5±0·9 | - | - |
| ETI therapy 1–3 months | | | –0·2±0·9 | 0·3 (0·2, 0·4) | <0·0001 |
| ETI therapy 4–6 months | | | –0·2±0·9 | 0·4 (0·3, 0·5) | <0·0001 |
| ETI therapy 7–9 months | | | –0·1±0·9 | 0·5 (0·4, 0·6) | <0·0001 |
| ETI therapy 10–12 months | | | –0·1±0·9 | 0.4 (0·3, 0·6) | <0·0001 |
| **PREVIOUS CFTR MODULATOR USE POPULATION (n=186)** | | | | | |
| **ppFEV_1_** | | |  |  |  |
| Previous 10–12 months | | | 61·6±23·2 | 0·6 (–0·7, 1·8) | 0·3840 |
| *Baseline* | | | 62·2±23·8 | - | - |
| ETI therapy 1–3 months | | | 72·1±24·7 | 9·9 (8·6, 11·2) | <0·0001 |
| ETI therapy 4–6 months | | | 72·5±24·5 | 10·3 (9·0, 11·6) | <0·0001 |
| ETI therapy 7–9 months | | | 73·1±24·6 | 10·9 (9·6, 12·3) | <0·0001 |
| ETI therapy 10–12 months | | | 72·7±24·4 | 10·5 (9·1, 11·9) | <0·0001 |
| **BMI (adults), kg/m^2^** | | |  |  |  |
| Previous 10–12 months | | | 20·9±3·1 | 0·3 (0·1, 0·4) | <0·0011 |
| *Baseline* | | | 21·2±3·2 | - | - |
| ETI therapy 1–3 months | | | 22·1±3·2 | 0·9 (0·7, 1·1) | <0·0001 |
| ETI therapy 4–6 months | | | 22·3±3·2 | 1·2 (1·0, 1·4) | <0·0001 |
| ETI therapy 7–9 months | | | 22·5±3·2 | 1·3 (1·1, 1·5) | <0·0001 |
| ETI therapy 10–12 months | | | 22·6±3·3 | 1·4 (1·2, 1·7) | <0·0001 |
| **BMI z-score (adolescents)** | | |  |  |  |
| Previous 10–12 months | | | ­0·8±0·8 | 0·1 (–0·0, 0·3) | 0·0522 |
| *Baseline* | | | –0·7±0·9 | - | - |
| ETI therapy 1–3 months | | | –0·5±0·8 | 0·2 (0·1, 0·3) | 0·0083 |
| ETI therapy 4–6 months | | | –0·4±0·8 | 0·3 (0·2, 0·5) | <0·0001 |
| ETI therapy 7–9 months | | | –0·5±0·9 | 0·2 (0·1, 0·4) | 0·0095 |
| ETI therapy 10–12 months | | | –0·5±0·8 | 0·2 (0·1, 0·4) | 0·099 |

BMI, body mass index; CI, confidence interval; ETI, elexacaftor/tezacaftor/ivacaftor; ppFEV_1_, percent predicted forced expiratory volume in 1 second; SD, standard deviation·

*Change is calculated as mean minus baseline mean except for the ‘Previous 10–12 months’ rows where change is calculated as baseline minus the mean for the 10–12 months before starting ETI·

**p-values were calculated using paired t-test and corrected using the Benjamini-Hochberg method

**Table S3. Absolute and relative frequencies of supplemental feeding before and after initiation of elexacaftor/tezacaftor/ivacaftor (ETI)**

| **Supplemental feeding (n=2313)** | **Before initiation of ETI** | **After initiation of ETI** |
| --- | --- | --- |
| Yes | 693 (30·0%) | 559 (24·2%) |
| No | 1620 (70·0%) | 1754 (75·8%) |

**Figure S1:** Number of people with cystic fibrosis (pwCF) starting treatment with elecacaftor/tezacaftor/ivacaftor (ETI) throughout the study period


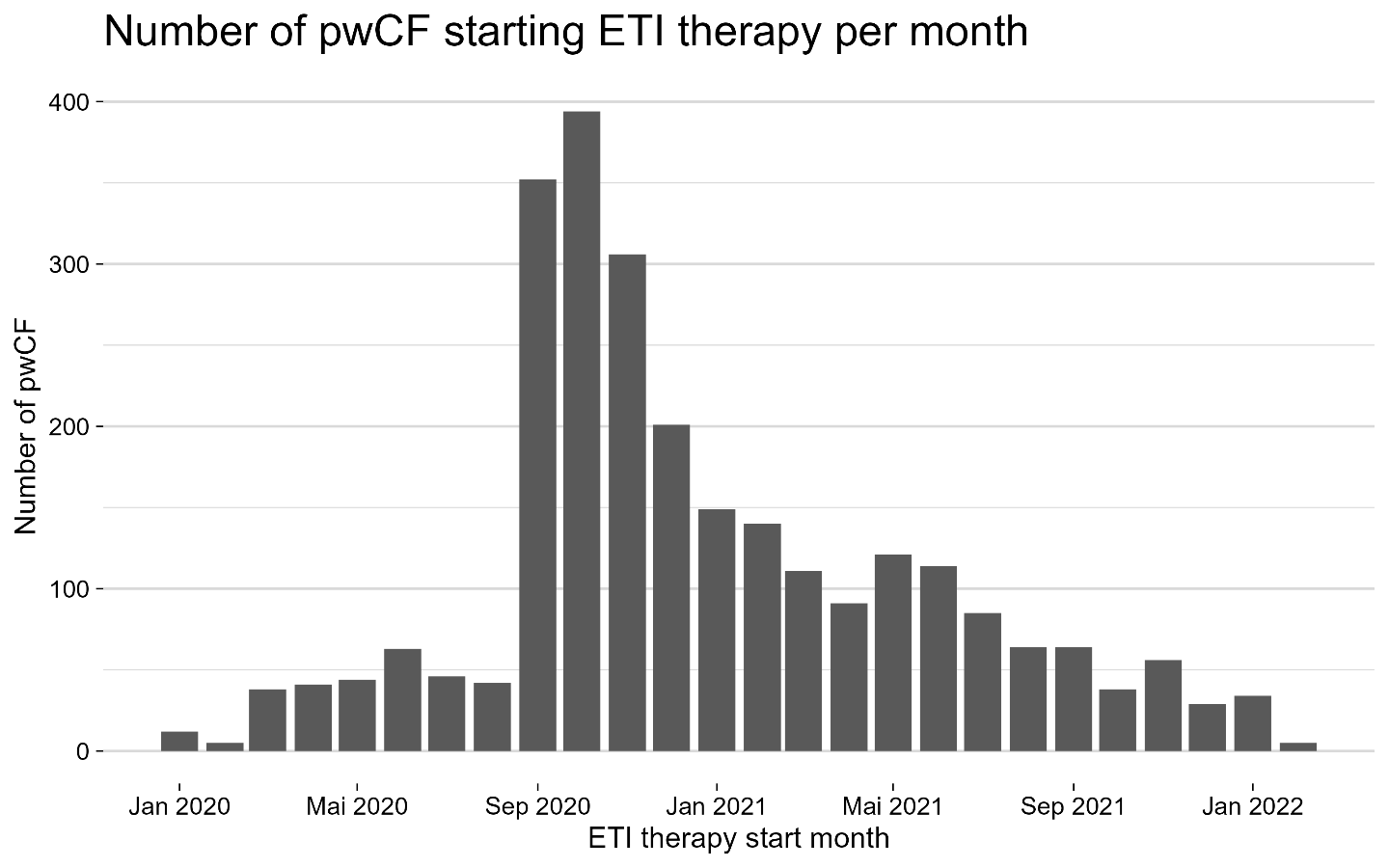


**Figure S2:** Change in percent predicted forced expiratory volume in 1 second (ppFEV_1_) over time before and after initiation of elecacaftor/tezacaftor/ivacaftor (ETI) therapy in participants with data available from every quarter.

pwCF, people with cystic fibrosis.


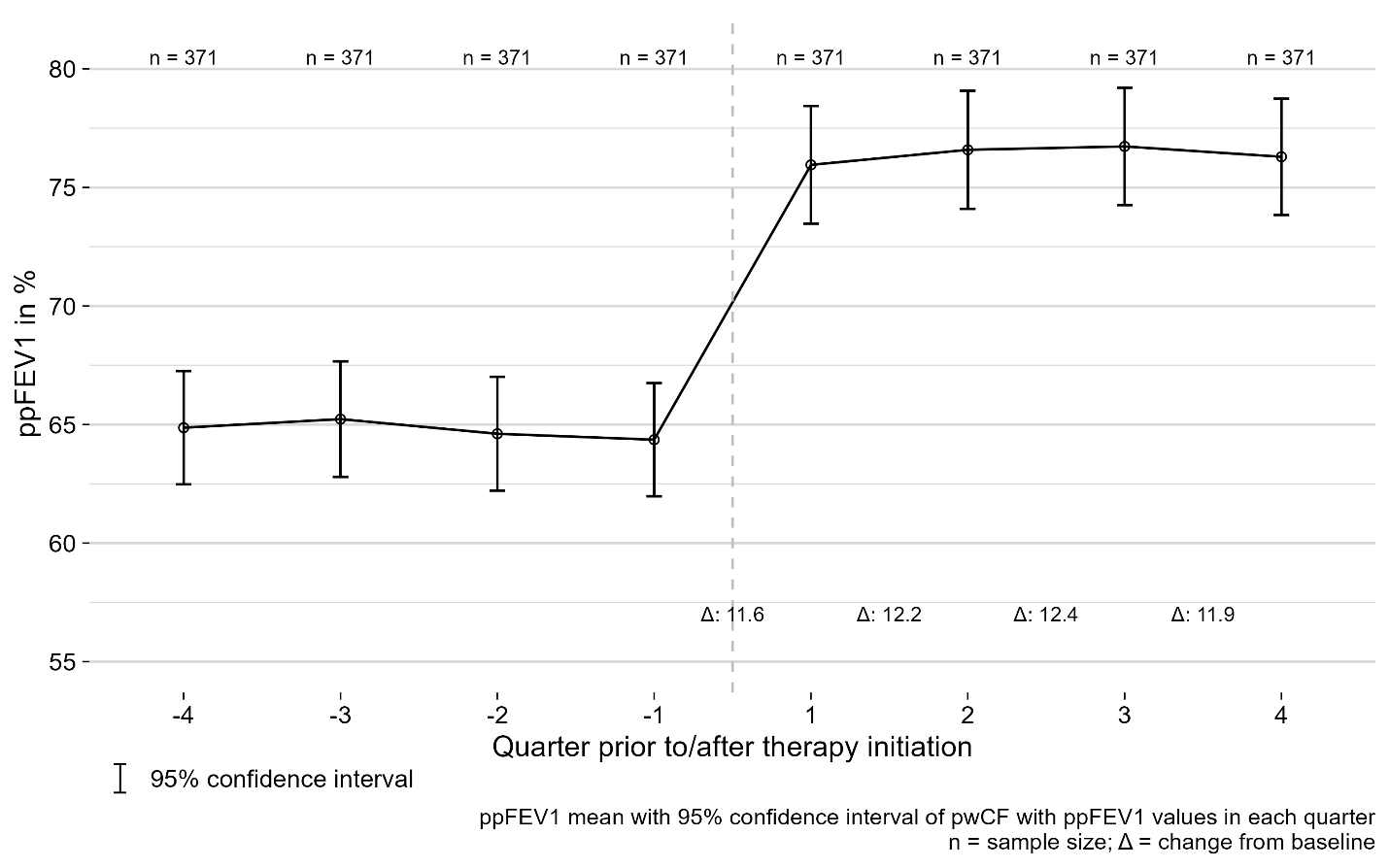


**Figure S3:** Change in percent predicted forced expiratory volume in 1 second (ppFEV_1_) over time before and after initiation of elecacaftor/tezacaftor/ivacaftor (ETI) therapy in males (dark blue) and females (magenta).


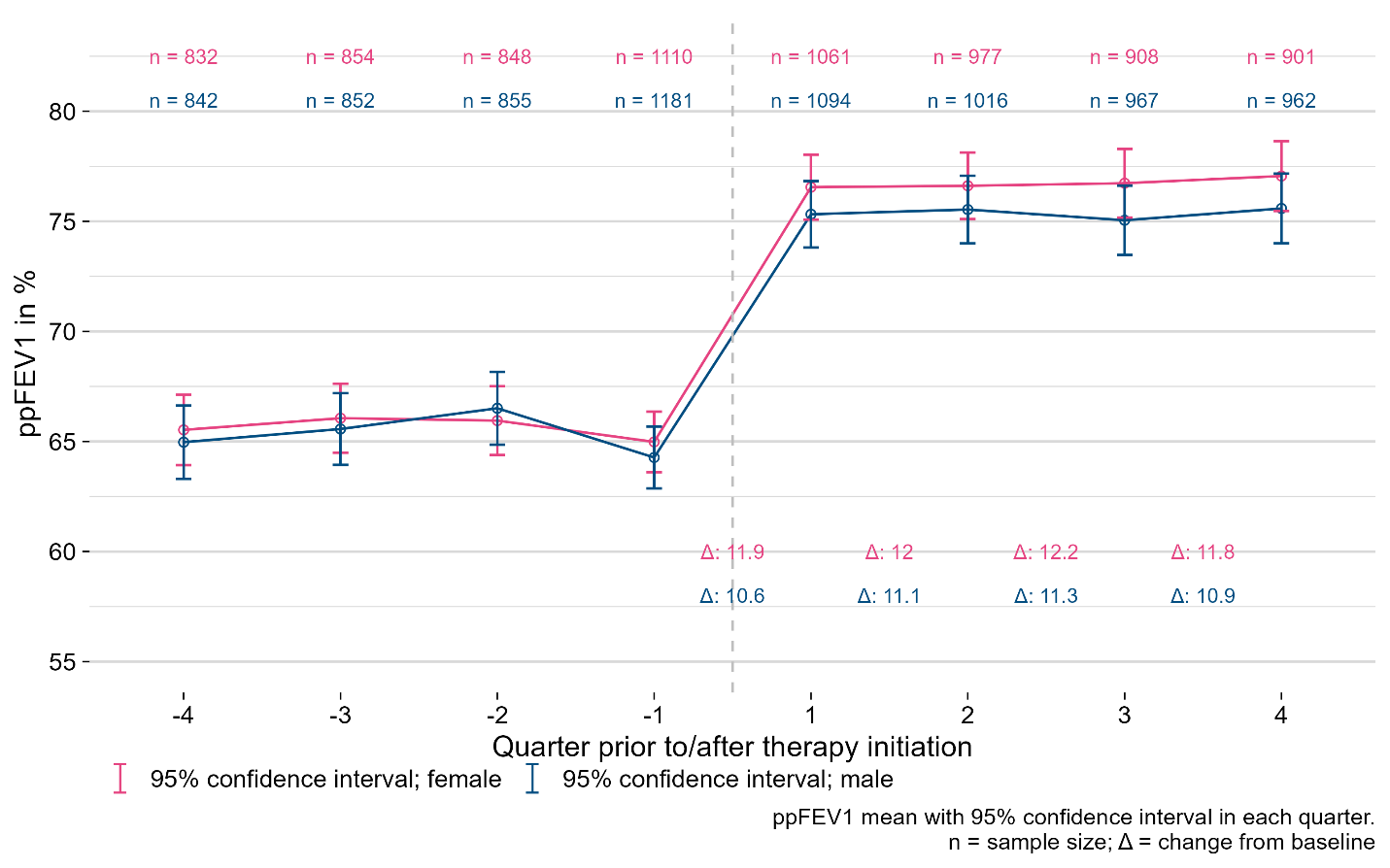


**Figure S4:** Change in body mass index (BMI) over time before and after initiation of elecacaftor/tezacaftor/ivacaftor (ETI) therapy in participants with data available from every quarter.

pwCF, people with cystic fibrosis.


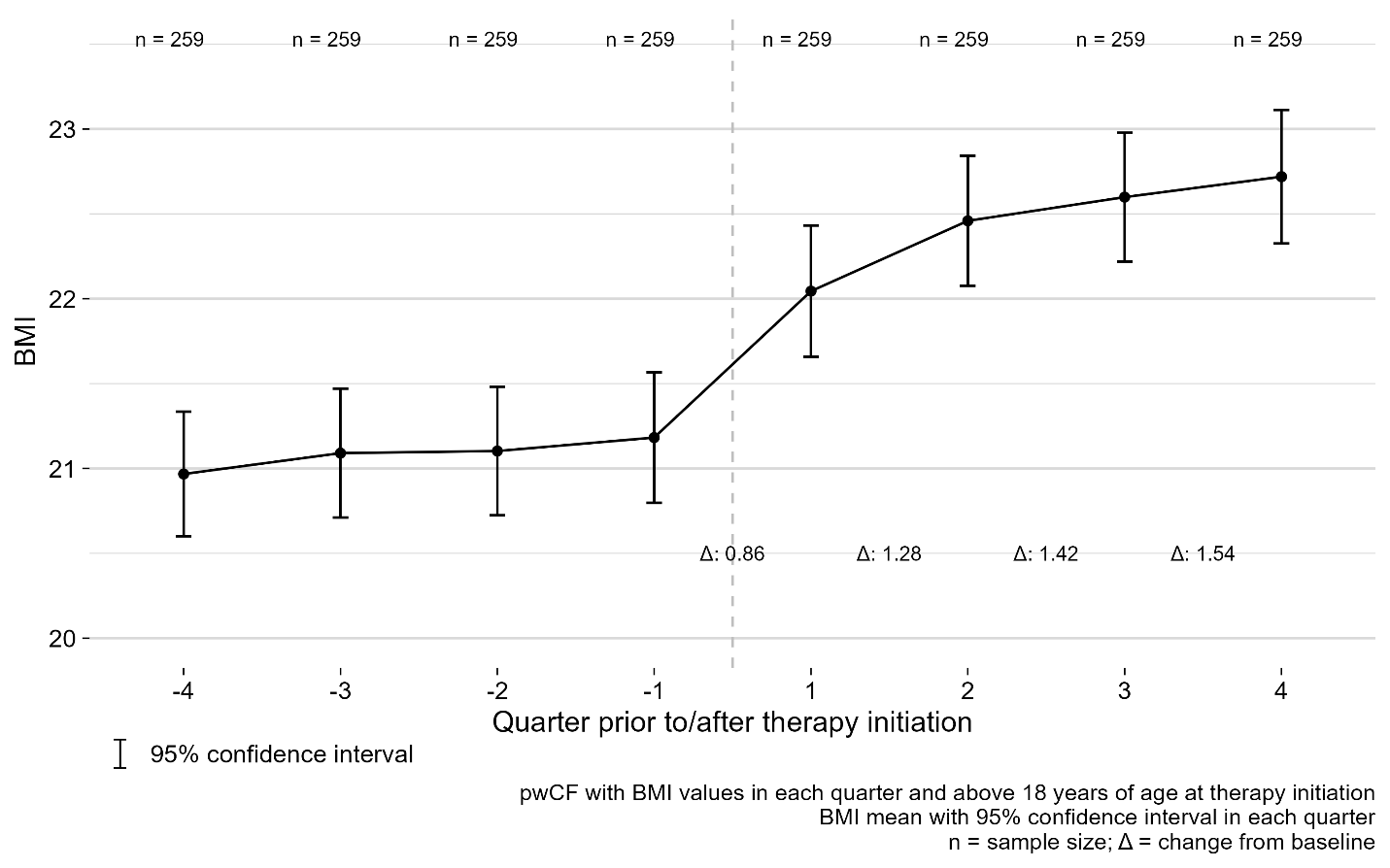


**Figure S5:** Change in body mass index (BMI) over time before and after initiation of elecacaftor/tezacaftor/ivacaftor (ETI) therapy in males (dark blue) and females (magenta).

pwCF, people with cystic fibrosis.


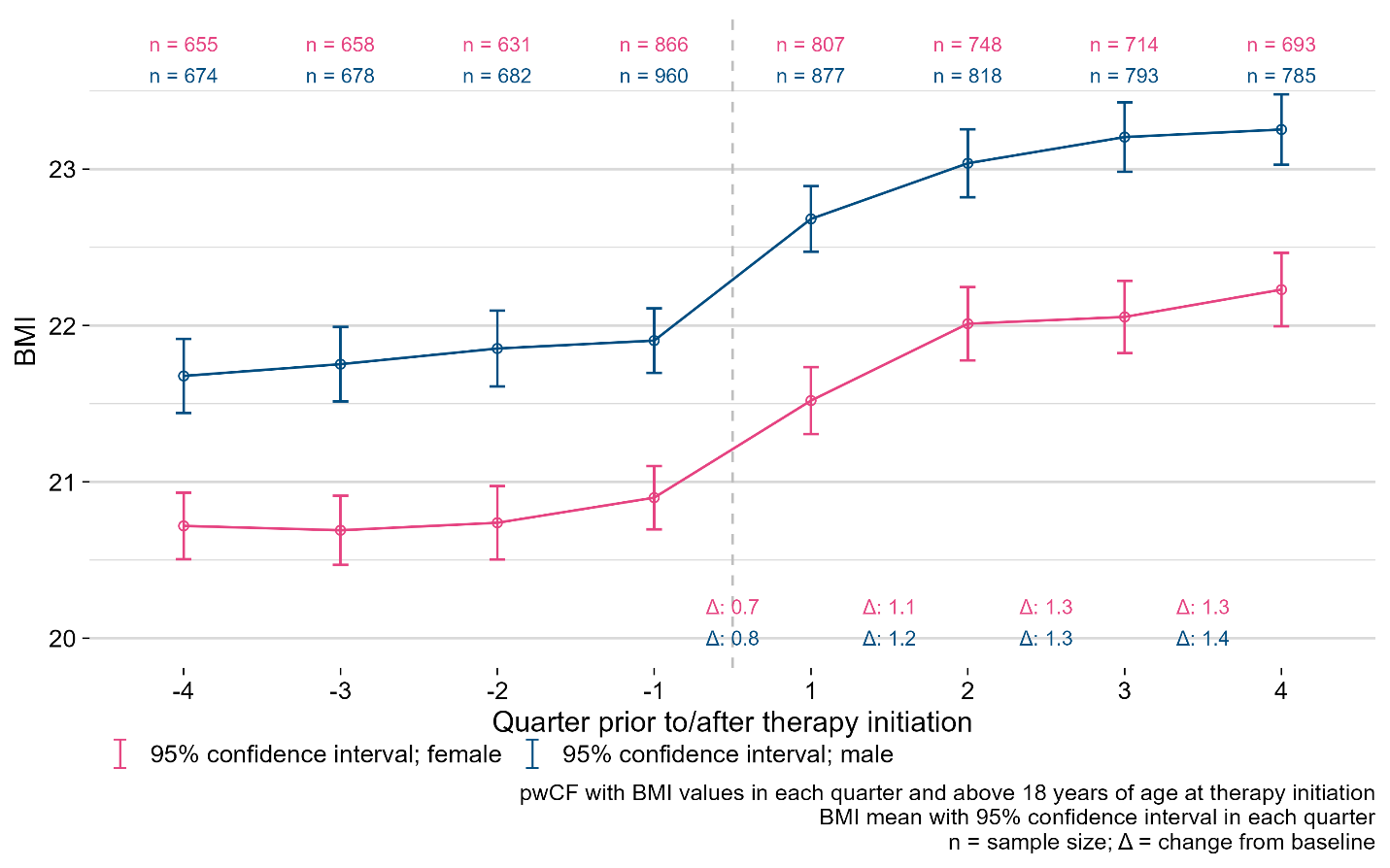


**Figure S6:** Change in body mass index (BMI) *z*-score over time before and after initiation of elecacaftor/tezacaftor/ivacaftor (ETI) therapy in participants with data available from every quarter.

pwCF, people with cystic fibrosis.

**
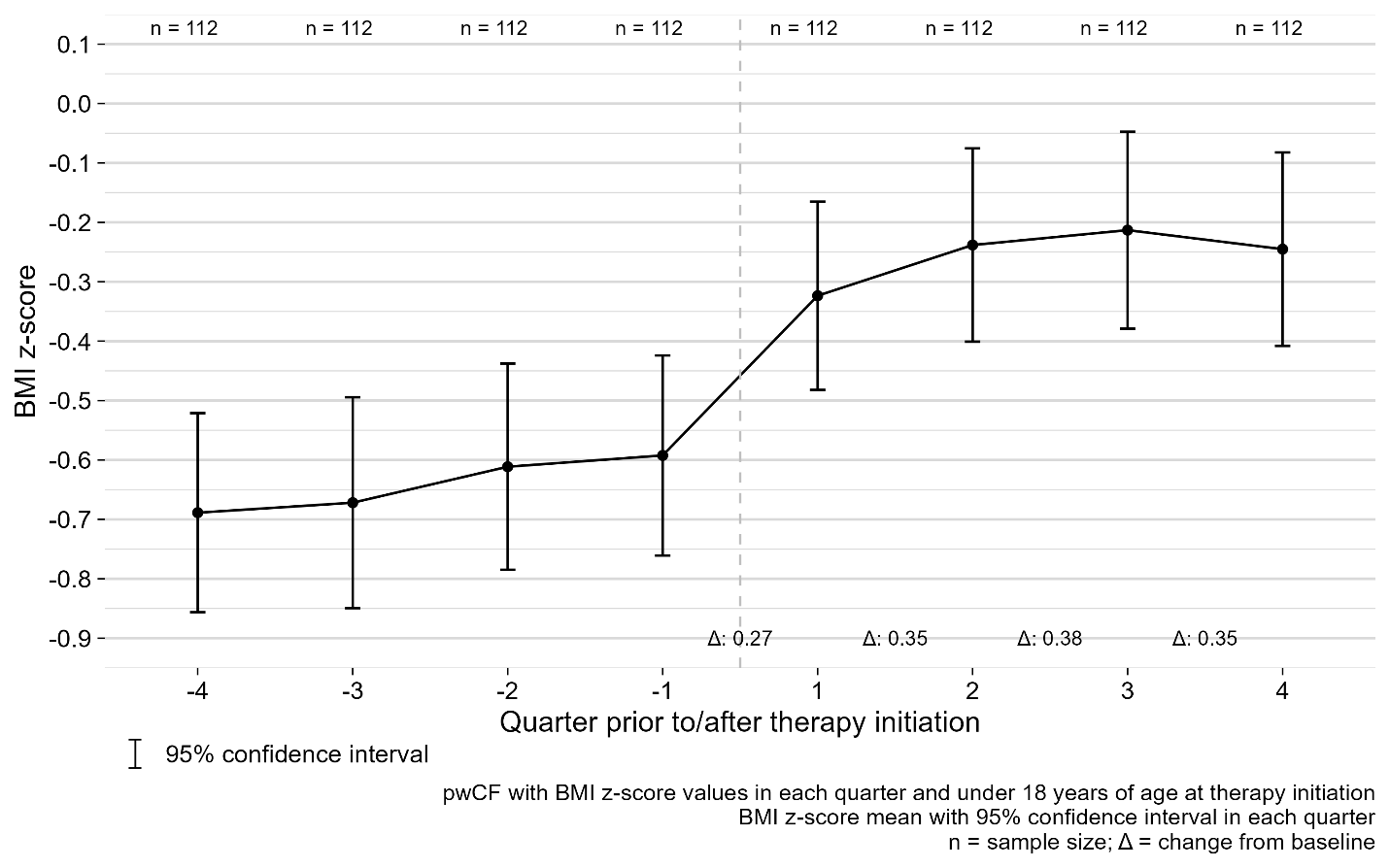
**

**Figure S7:** Change in body mass index (BMI) *z*-score over time before and after initiation of elecacaftor/tezacaftor/ivacaftor (ETI) therapy in males (dark blue) and females (magenta).

pwCF, people with cystic fibrosis.

**
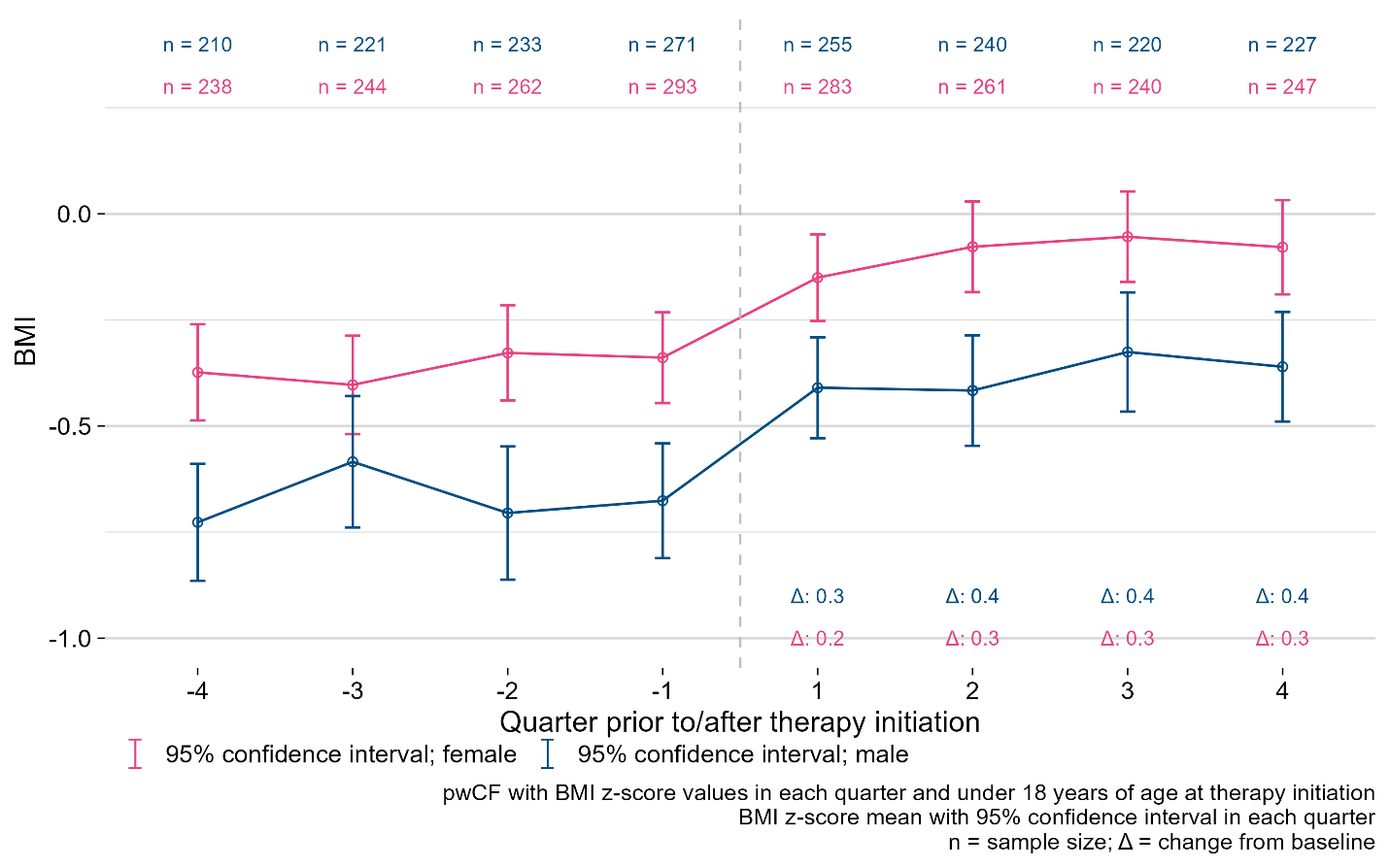
**

**Figure S8** Change in total number of sweat chloride in the 12 months before and after initiation of elecacaftor/tezacaftor/ivacaftor (ETI) therapy in people with cystic fibrosis


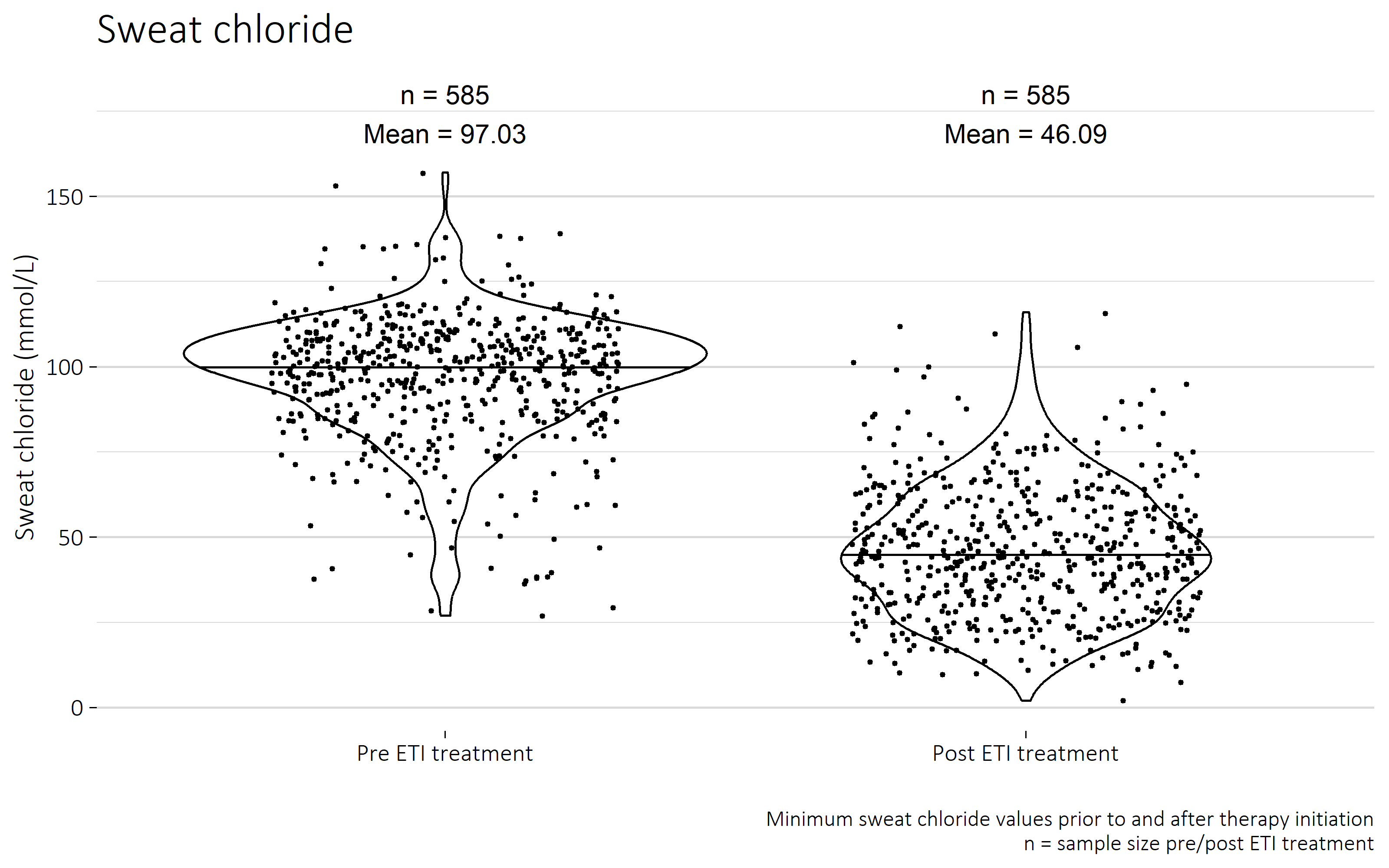


**Figure S9** Change in **s**weat chloride pre and post ETI treatment by gender


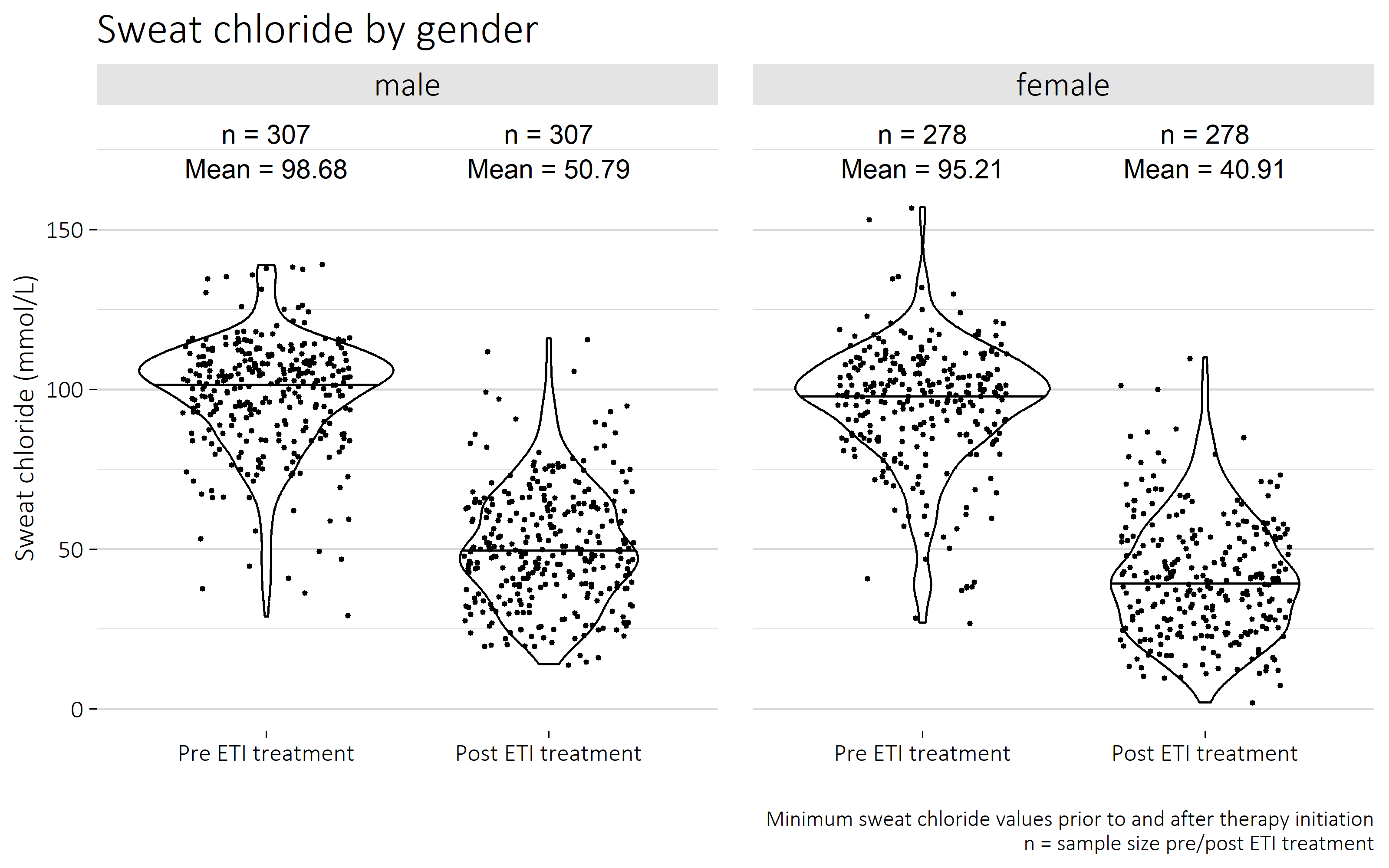


**Figure S10:** Change in total number of exacerbations in the 12 months before and after initiation of elecacaftor/tezacaftor/ivacaftor (ETI) therapy in people with cystic fibrosis who had examinations in both the year before and the year after therapy initiation


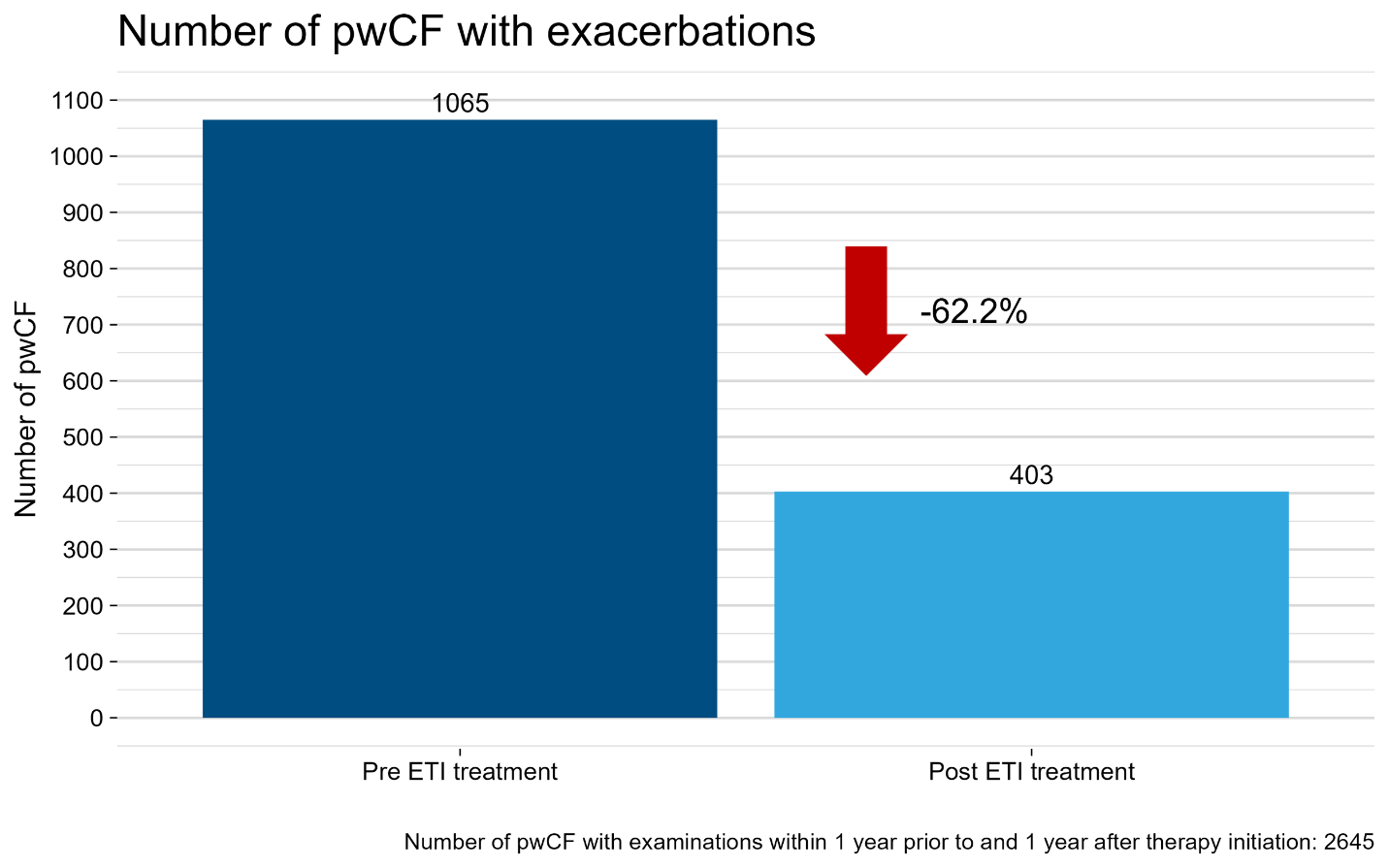


**Figure S11:** Number of people with cystic fibrosis (pwCF) experiencing exacerbations in each quarter before and after initiation of elecacaftor/tezacaftor/ivacaftor (ETI) therapy


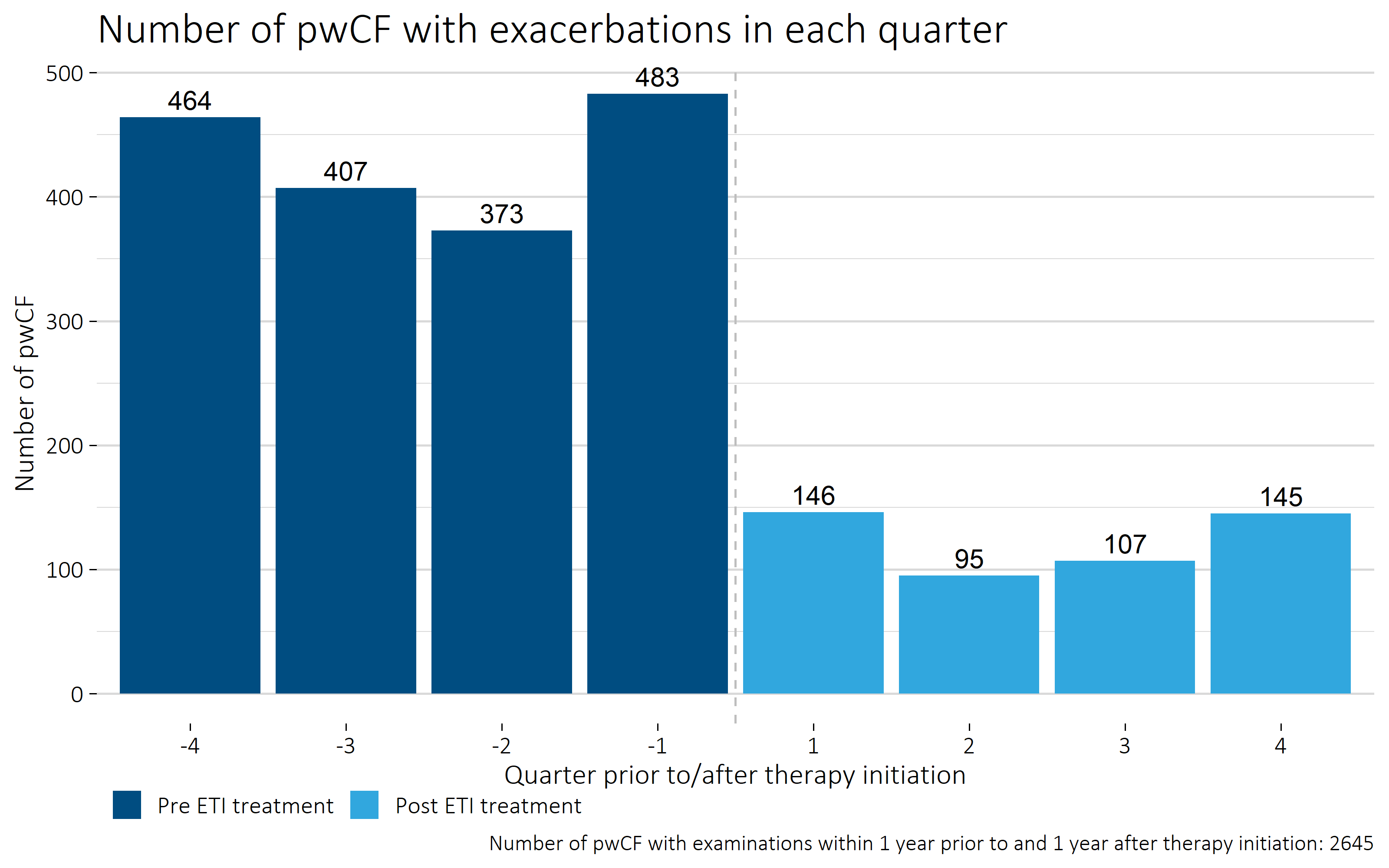

Supplement: Supplementary Tables Figures [file mmc1.docx]
