# Supplementary material for: Impact of elexacaftor/tezacaftor/ivacaftor on lung function, nutritional status, pulmonary exacerbation frequency and sweat chloride in people with cystic fibrosis: real-world evidence from the German CF Registry
Source: Lancet Reg Health Eur. 2023 Jul 28;32:100690. doi: 10.1016/j.lanepe.2023.100690 (PMC10405057; doi:10.1016/j.lanepe.2023.100690)
Supplement: Appendix List [file mmc2.docx]

**List of centres participating in the German CF registry (2021)**

| Title | Surname | Last Name | Department | Hospital Name/Faculty |
| --- | --- | --- | --- | --- |
| PD. Dr. | Klaus | Tenbrock | Klinik für Kinder- und Jugendmedizin Pädiatrische Pneumologie | Uniklinik RWTH Aachen |
| Dr. | Claus | Pfannenstiel |  | Kinderarztpraxis Laurensberg Aachen |
| Dr. | Dirk | Steffen | Innere Medizin | Luisenhospital Aachen |
| Dr. | Jochen | Meister | Klinik für Kinder- und Jugendmedizin | HELIOS Klinikum Aue |
| Dr. | Britta | Welzenbach | Klinik für Kinder- und Jugendmedizin Sozialpädagogisches Zentrum (SPZ) | KJF Klinik Josefinum Augsburg |
| Dr. | Anette | Scharschinger | Klinik für Kinder- und Jugendmedizin Kinderpneumologie und Allergologie | Universitätsklinikum Augsburg |
| Dr. | Markus | Kratz | Klinik für Kinder- und Jugendmedizin Baden-Baden Balg | Klinkum Mittelbaden gGmbH, Baden-Baden |
| Dr. | Maike | Pincus | Klinik für Kinder- und Jugendmedizin Pulmologie und Allergologie | Helios Klinikum Berlin Buch, Berlin |
| Prof. Dr. | Tobias | Tenenbaum | Klinik für Kinder- und Jugendmedizin | Sana Klinkum Lichtenberg, Berlin |
| Prof. Dr. | Mirjam | Stahl | Klinik für Pädiatrie m. S. Pneumologie und Immunologie - Christiane Herzog-Zentrum | Charité - Universitätsmedizin Berlin |
| Dr. | Kerstin | Landwehr | Klinik für Kinder- und Jugendmedizin Schwerpunkt Kinder-Pneumologie | Evangelisches Klinikum Bethel, Bielefeld |
| Dr. | Stefanie | Dillenhöfer | St. Josef Hospital - Klinik für Kinder- und Jugendmedizin - Christiane Herzog Zentrum | UKRUB - Katholisches Klinikum Bochum |
| Dr. | Hans | Kössel | Klinikum Westbrandenburg Pädiatrische Pulmologie und Allergologie | Medizinische Hochschule Brandenburg (MHB) |
| Dr. | Petra | Kaiser | Klinik für Kinder- und Jugendmedizin Christiane Herzog-Zentrum | Eltern-Kind-Zentrum Prof. Hess, Bremen |
| Dr. | Manfred | Käding | Praxis für Kinder- und Jugendmedizin | Poliklinik Chemnitz |
| Dr. | Simone | Stolz | Akademisches Lehrkrankenhaus der Charité Klinik für Kinder- und Jugendmedizin | Carl-Thiem-Klinikum Cottbus |
| Dr. | Stefan | Blaas | Zentrum für Pneumologie | Klinik Donaustauf |
| Dr. | Jutta | Hammermann | Haus 21 Universitäts-Mukoviszidose-Centrum (UMC) | Universitätsklinikum Carl Gustav Carusc, Dresden |
| Prof. Dr. | Monika | Gappa | Klinik für Kinder und Jugendliche Kinderpneumologie und Allergologie | Evangelisches Krankenhaus Düsseldorf |
| Prof. Dr. | Antje | Schuster | Klinik für Allgemeine Pädiatrie, Neonatologie und Kinderkardiologie | UKD Universitätsklinikum Düsseldorf |
| Dr. | Dana | Spittel | Kinder- und Jugendmedizin | Helios Klinikum Erfurt |
|  | Sabine | Zirlik | Medizinische Klinik 1 Schwerpunkt Pneumologie | Universitätsklinikum Erlangen |
| PD Dr. | Sabina | Schmitt | Kinderklinik - Sozialpädiatrisches Zentrum | Universitätsklinikum Erlangen |
| PD Dr. | Florian | Stehling | Klinik für Kinderheilkunde III Christiane Herzog Centrum Ruhr | Universitätsklinikum Essen (AöR) |
| Dr. | Sivagurunathan | Sutharsan | Ruhrlandklinik - Klinik für Pneumologie, Christiane Herzog-Zentrum | Universitätsmedizin Essen |
| Prof. Dr. | Joachim | Bargon | Klinik für Pneumologie | Frankfurter Rotkreuz-Kliniken e.V., Frankfurt |
| PD Dr. | Malte | Cremer | Zentrum für Kinder- und Jugendmedizin, Allgemeine Pädiatrie | Klinikum Frankfurt (Oder) |
| Dr. | Christina | Smaczny | Klinik für Kinder- und Jugendmedizin, Christiane Herzog CF-Zentrum | Universitätsklinikum Frankfurt |
| Dr. | Sebastian | Fähndrich | Klinik für Pneumologie | Universitätsklinikum Freiburg |
| Prof. Dr. | Andrea | Heinzmann | Zentrum für Kinder- und Jugendmedizin | Universitätsklinikum Freiburg |
| Prof. Dr. | Lutz | Nährlich | Zentrum für Kinderheilkunde und Jugendmed. Abteilung Allgemeine Pädiatrie und Neonatologie | Universitätsklinikum Gießen-Marburg GmbH |
| Dr. | Stefan | Kuhnert | Medizinische Klinik und Poliklinik II | Universitätsklinikum Gießen |
| PD Dr. | Sebastian | Schmidt | Klinik für Kinder- und Jugendmedizin | Universitätsmedizin Greifswald |
| Dr. | Bettina | Wollschläger | Medizinische Fakultät der Klinik für Innere Medizin I | Universitätsklinikum Halle (Saale) UKH, Halle |
| Dr. | Anna | Nolde | II. Medizinische Klinik und Poliklinik Sektion Pneumologie | Universitätsklinikum Eppendorf UKE, Hamburg, |
| Dr. | Inka | Held | Praxis Kinderärzte im Friesenweg CF Centrum Altona | Kinder- und Jugendärztliche Gemeinschaftspraxis, Hamburg |
| Prof. Dr. | Wolfgang | Kamin | Klinik für Kinder- und Jugendmedizin Pulmologie/Allergologie | Evangelisches Krankenhaus Hamm (EVK) |
| PD Dr. | Felix C. | Ringshausen | Klinik für Pneumologie | Medizinische Hochschule Hannover (MHH) |
| Prof. Dr. | Anna-Maria | Dittrich | Klinik für Pädiatrische Pneumologie Christiane Herzog-Zentrum | Medizinische Hochschule Hannover (MHH) |
| Dr. | Sabine | Wege | Thoraxklinik Heidelberg gGmbH Pneumologie und Beatmungstherapie | Universitätsklinik Heidelberg |
| PD Dr. | Olaf | Sommerburg | Sektion Pädiatrische Pneumologie und Allergologie | Universitätsklinikum Heidelberg |
| Dr. med. | Norbert | Geier | Klinik für Kinder- und Jugendmedizin Klinikum am Gesundbrunnen, Perinatalzentrum | SLK-Kliniken Heilbronn |
|  | Sara Lisa | Fleser | Klinik für Allgemeine Pädiatrie und Neonatologie | Universitätsklinikum des Saarlandes, Homburg |
| Prof. Dr. | Heinrike | Wilkens | Innere Medizin V - Pneumologie, Allergologie Beatmungs- und Umweltmedizin | Universitätsklinikum des Saarlandes. Homburg |
| Prof. Dr. | Helmut | Ellemunter | Tirol Kliniken GmbH Kinder- und Jugendheil- kunde - Mukoviszidose-Zentrum | Medizinische Universität Innsbruck |
|  | Michael | Lorenz | Klinik für Kinder- und Jugendmedizin | Universitätsklinikum Jena |
| Dr. | Paul | Vöhringer | Klinik für Kinder- und Jugendmedizin Pneumologie | Städtisches Klinikum Karlsruhe |
| Dr. | Martin | Schebek | Pädiatrische Hämatologie und Onkologie, Psychosomatik und Systemerkrankungen | Klinikum Kassel |
| Dr. | Christian | Timke | Kinderklinik und Jugendmedizin Christiane Herzog Zentrum Nord | Städtisches Krankenhaus Kiel |
| Dr. | Ingrid | Bobis | 4. Medizinische Klinik Christiane Herzog Zentrum Nord | Städtisches Klinikum Kiel |
| PD Dr. | Thomas | Nüßlein | Klinik für Kinder- und Jugendmedizin | Gemeinschaftsklinikum Mittelrhein, Koblenz |
| Dr. | Doris | Dieninghoff | Lungenklinik Merheim | Kliniken der Stadt Köln |
| PD Dr. | Ernst | Rietschel | Klinik für Kinder- und Jugendmedizin | Universitätsklinikum Köln |
|  | Bastian | Klinkhammer | Zentrum für Kinder- und Jugendmedizin | Helios Klinikum Krefeld |
| PD Dr. | Freerk | Prenzel | Klinik und Poliklinik für Kinder- und Jugendmedizin | Universitätsklinikum Leipzig |
| Dr. | Alexandra | Wald | Klinik und Poliklinik für Pneumologie | Universitätsklinikum Leipzig |
| Dr. | Axel | Kempa | Fachklinik Löwenstein | SLK-Kliniken Heilbronn, Löwenstein |
| Prof. Dr. | Folke | Brinkmann | Campus Lübeck Klinik für Kinder- und Jugendmedizin | Universitätsklinikum Schleswig Holstein, Lübeck |
| Dr. | Eva | Lücke | Klinik für Pneumologie | Otto-von-Guericke-Universität Magdeburg |
|  | Ines | Adams | Kinderklinik | Otto-von-Guericke Universität Magdeburg |
| Dr. | Krystyna | Poplawska | Klinik für Kinder- und Jugendmedizin Pädiatrische Pneumologie und Allergologie | Universitätsmedizin Mainz |
| Dr. | Simone | Lehmkühler | Klinik für Kinder- und Jugendmedizin Pädiatrische Pneumologie und Allergologie | Universitätsmedizin Mannheim |
| Dr. | Monika | Bauck | Klinik für Kinder- und Jugendmedizin II Pädiatrische Pneumologie | Phillipps Universität Marburg |
|  | Anne | Pfülb | Klinik für Kinder- und Jugendmedizin | Klinikum Memmingen |
| Prof. Dr. | Rainald | Fischer |  | Lungenheilkunde München Pasing, München |
| Dr. | Gudrun | Schopper | Klinik für Kinder- und Jugendmedizin Allergologie, Pneumologie, Umweltmedizin | München Klinik Schwabing, München |
| Dr. | Susanne | Nährig | Campus Innenstadt Medizinische Klinik - Pneumologie | LMU Klinikum der Universität München |
| Prof. Dr. | Matthias | Griese | Kinderklinik und Kinderpoliklinik im Dr. von Haunerschen Kinderspital | LMU Klinikum der Universität München |
| Dr. | Jörg | Grosse | Klinik für Kinder- und Jugendmedizin Allgemeine Pädiatrie | Universitätsklinikum Münster UKM |
| Dr. med. | Peter | Küster | Kinder- und Jugendmedizin | Clemenshospital Münster |
| Dr. | Birte | Kinder | Klinik für Kinder- und Jugendmedizin | Dietrich Bonhoeffer Klinikum, Neubrandenburg |
| Dr. | Holger | Köster | Klinik für Pädiatrische Pneumologie und Allergologie, Neonatologie und Intensivmedizin | Klinikum Oldenburg AöR |
| Dr. | Susanne | Büsing | Zentrum für Kinder- und Jugendmedizin | Christliches Kinderhospital Osnabrück |
|  | Margarethe | Pohl | Zentrum für Kinder- und Jugendgesundheit Kinderpneumologie | Kinderklinik Dritter Orden, Passau |
| Prof. Dr. | Carsten | Schwarz | Kinder- und Jugendklinik | Klinikum Westbrandenburg gGmbH, Potsdam |
| PD Dr. | Andreas | Artlich | Klinik für Kinder und Jugendliche | Oberschwabenklinik (OSK) gGmbH, Ravensburg |
| Dr. | Alexander | Kiefer | Kinder- und Jugendmedizin | Klinik St. Hedwig, Regensburg |
| Prof. Dr. | Manfred | Ballmann | Kinder- und Jugendklinik Pneumologie und Allergologie | Universitätsmedizin Rostock |
| Dr. | Nikola | Gjorgjevski | Kinder- und Jugendmedizin | Helios Kliniken Schwerin |
| Prof. Dr. | Markus A. | Rose | Zentrum für chronische Lungenerkrankungen Christiane Herzog Transitionszentrum | Klinikum Stuttgart – Olgahospital, Stuttgart |
| Dr. | Friederike | Ruf | Pneumologie und Beatmungsmedizin | Robert Bosch Krankenhaus RBK, Stuttgart |
| Dr. | Rolf | Mahlberg | Innere Medizin 1 | Klinikum Mutterhaus der Borromäerinnen, Trier |
| PD Dr. | Wolfgang | Thomas | Kinder- und Jugendmedizin | Klinikum Mutterhaus der Borromäerinnen, Trier |
| Dr. | Ute | Graepler | Klinik für Kinder- und Jugendmedizin | Universitätsklinikum Tübingen |
| PD Dr. | Sebastian | Bode | Klinik für Kinder- und Jugendmedizin | Universitätsklinikum Ulm |
| Dr. | Philipp | Meyn | Fachkliniken Wangen Klinik für Pneumologie | Waldburg Zeil Kliniken, Wangen |
| Prof. Dr. | Josef | Rosenecker | Fachkliniken Wangen Rehabilitationsklinik für Kinder und Jugendliche | Waldburg-Zeil Kliniken, Wamgen |
| Dr. | Cordula | Koerner | Klinik für Kinder- und Jugendmedizin | Marien Hospital Wesel gGmbH |
| Prof. Dr. | Klaus-Michael | Keller | Klinik für Kinder- und Jugendmedizin | DKD Helios Klinik Wiesbaden |
| Dr. | Tina | Teßmer | Klinik für Kinder- und Jugendmedizin | Klinikum Worms gGmbH |
| Prof. Dr. | Helge | Hebestreit | Kinderpoliklinik Christiane Herzog-Zentrum Unterfranken | Universitätsklinikum Würzburg |
| Dr. | Gerhild | Lohse | Universitätsklinik und Poliklinik für Kinder und Jugendliche | Heinrich Braun Klinikum gGmbH, Zwickau |
